# A laboratory ice machine as a cold oligotrophic artificial microbial niche for biodiscovery

**DOI:** 10.1038/s41598-023-49017-0

**Published:** 2023-12-12

**Authors:** Leila Satari, Daniel Torrent, Asier Ortega-Legarreta, Adriel Latorre-Pérez, Javier Pascual, Manuel Porcar, Alba Iglesias

**Affiliations:** 1 Institute for Integrative Systems Biology (I2SysBio), University of Valencia-CSIC, Paterna, Spain; 2 Darwin Bioprospecting Excellence S.L., Paterna, Spain

**Keywords:** Biofilms, Microbial communities

## Abstract

Microorganisms are ubiquitously distributed in nature and usually appear as biofilms attached to a variety of surfaces. Here, we report the development of a thick biofilm in the drain pipe of several standard laboratory ice machines, and we describe and characterise, through culture-dependent and -independent techniques, the composition of this oligotrophic microbial community. By using culturomics, 25 different microbial strains were isolated and taxonomically identified. The 16S rRNA high-throughput sequencing analysis revealed that *Bacteroidota* and *Proteobacteria* were the most abundant bacterial phyla in the sample, followed by *Acidobacteriota* and *Planctomycetota*, while ITS high-throughput sequencing uncovered the fungal community was clearly dominated by the presence of a yet-unidentified genus from the *Didymellaceae* family. Alpha and beta diversity comparisons of the ice machine microbial community against that of other similar cold oligotrophic and/or artificial environments revealed a low similarity between samples, highlighting the ice machine could be considered a cold and oligotrophic niche with a unique selective pressure for colonisation of particular microorganisms. The recovery and analysis of high-quality metagenome-assembled genomes (MAGs) yielded a strikingly high rate of new species. The functional profiling of the metagenome sequences uncovered the presence of proteins involved in extracellular polymeric substance (EPS) and fimbriae biosynthesis and also allowed us to detect the key proteins involved in the cold adaptation mechanisms and oligotrophic metabolic pathways. The metabolic functions in the recovered MAGs confirmed that all MAGs have the genes involved in psychrophilic protein biosynthesis. In addition, the highest number of genes for EPS biosynthesis was presented in MAGs associated with the genus *Sphingomonas*, which was also recovered by culture-based method. Further, the MAGs with the highest potential gene number for oligotrophic protein production were closely affiliated with the genera *Chryseoglobus* and *Mycobacterium*. Our results reveal the surprising potential of a cold oligotrophic microecosystem within a machine as a source of new microbial taxa and provide the scientific community with clues about which microorganisms are able to colonise this ecological niche and what physiological mechanisms they develop. These results pave the way to understand how and why certain microorganisms can colonise similar anthropogenic environments.

## Introduction

Microorganisms are able to attach to surfaces and grow in biofilms. In fact, biofilms are the preferred mode of growth lifestyle for many microorganisms, as these strong and dynamic structures provide numerous advantages. Indeed, it has been calculated that as much as 40–80% of cells on our planet reside within biofilms^1^. Biofilms are ubiquitously distributed in nature, from marine and continental waters to rocks, plant surfaces and artificial structures, and include both microbial cells and a complex extracellular polymeric substance (EPS) matrix made of polysaccharides, secreted enzymes, amphiphilic compounds, and other macromolecules^2^. Microorganisms living in biofilms are found attached to a surface (rock, sand particles, metallic surfaces, plastic pipes, etc.) rather than following a free planktonic growth. In comparison to their suspended counterparts, biofilm-associated cells count with enhanced adhesion/cohesion capabilities, nutritional sources and a vast metabolite exchange network for communication, protection, and joint defence^3^, although they also display a reduced growth rate, and a different regulation of specific genes^4^.

Environmentally, massive overgrowth of biofilms can lead to serious disturbances in the flow of water streams or biological fluids. Biofilms are well known for their ability to overgrow and clog artificial substrates such as intermittent sand filters, in which clogging as a consequence of heterotrophs growth occurred in the upper part of the system^5^. They can also be responsible for the clogging of water distribution systems in drip irrigation emitters of treated wastewater^6^; the obstruction and contamination of machines such as reverse-osmosis water systems used for haemodialysis^7^; or cause runability problems in paper machines^8^. Biofilm development in artificial environments and mechanical devices, particularly those with high water loads, can yield to the risk of contamination by pathogens, for example in dishwashers^9,10^. Paradoxically, though, selected microbial populations can also be used to treat the effluents of those machines, such as the biofilter recently developed to treat dishwasher wastewater^11^. Biofilms are also found in industrial and domestic washing machines, where, interestingly, the majority of the microbial strains isolated formed substantial biofilms in washing machines worldwide^12^.

In 2019, an ice machine of a molecular biology laboratory in Valencia, Spain, suffered from several episodes of water leakage because of clogging of its water pump. Later, two other machines of the same Institute (in different floors) started displaying similar microbial growth and consequent water leaks. In all cases, a thick biofilm was found to totally clog the water reservoir of the water pump and part of the wastewater pipes. The biofilm was sampled and subjected to the complete study of its microbial composition that we report here.

## Results

**Figure 1.**
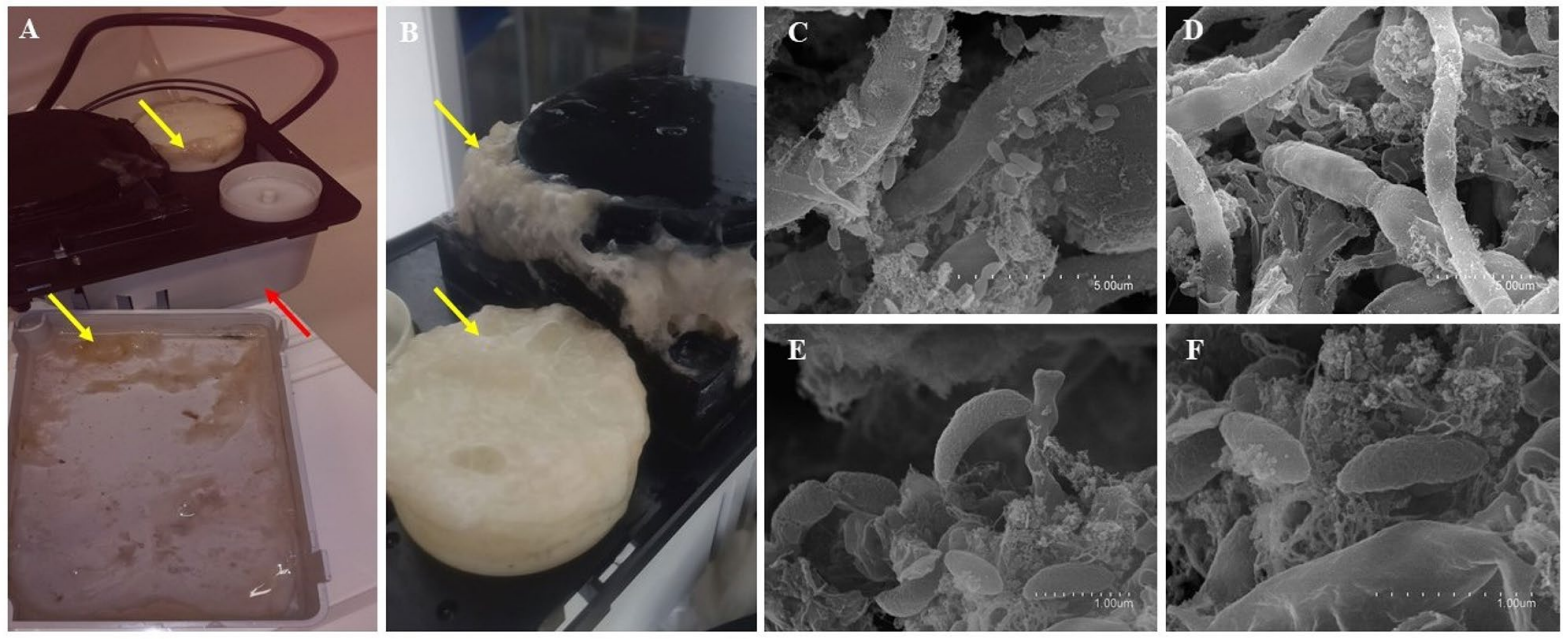
(**A**,**B**) The biofilm formed around the out-flux pipes of the laboratory ice machine. Yellow arrows point to the biofilm, and red arrows to the ice machine water reservoir. (**C**–**F**) The images from the Field Emission Scanning Electron Microscope (FE-SEM) of the biofilm show microbial cells within the EPS matrix.

In this work, we describe the microbial composition of a biofilm that colonised the drain pipe of laboratory ice machines. SEM analysis of the biofilm revealed a mixed community of bacteria and fungi embedded in an EPS-like matrix (Fig. 1A–F). Thus, both culturomics and metataxonomic analysis focused on the taxonomic identification and functional characterisation of the microorganisms involved in biofilm formation.

### Strain collection

**Figure 2:**
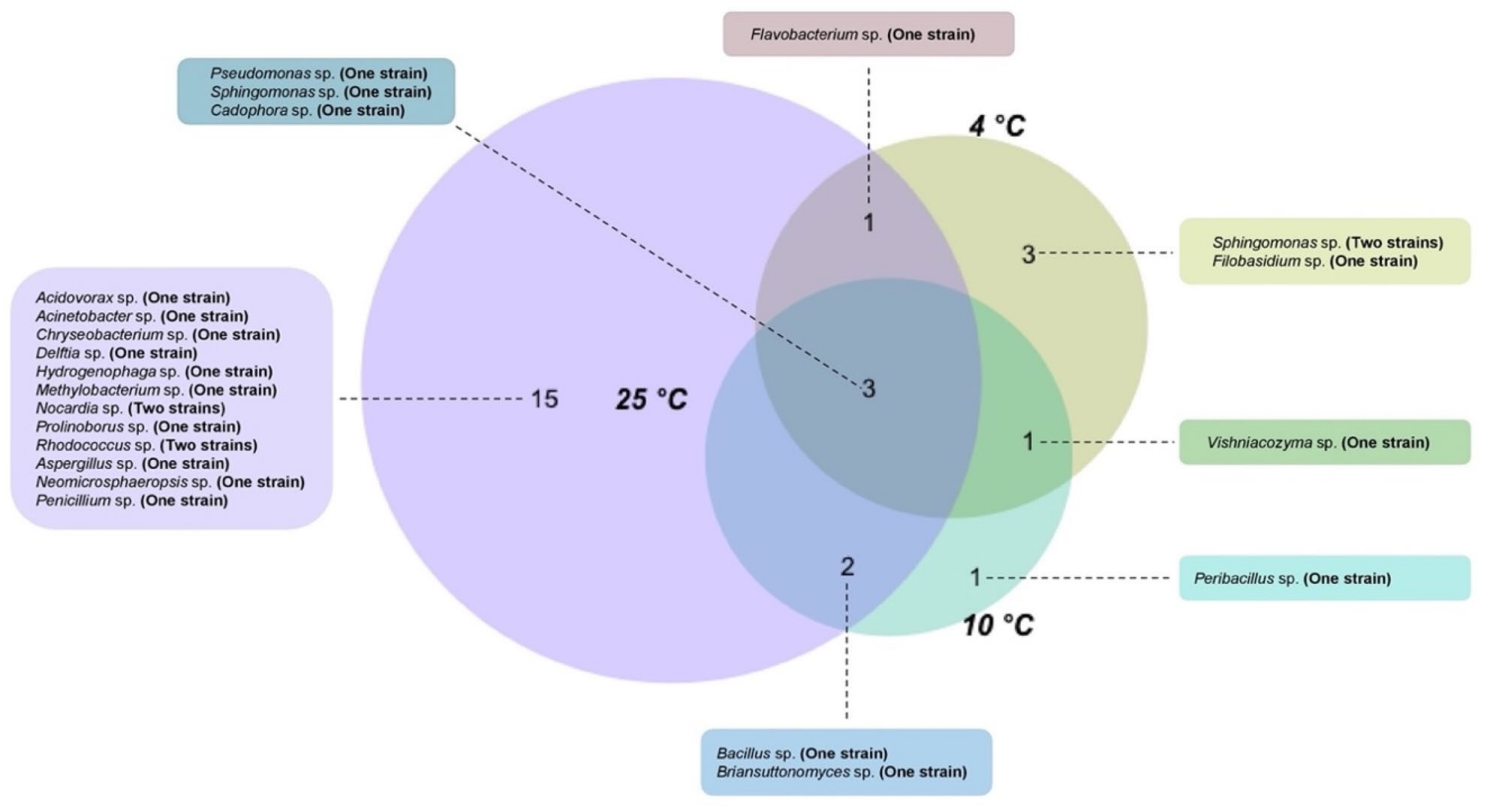
Venn diagram displaying the shared and unique isolated microorganisms among the three different temperatures. A higher diversity of isolates was observed at 25 °C compared to the other two incubating temperatures.

A set of 25 microbial strains (18 bacterial and seven fungal strains) were isolated and taxonomically identified from the ice machine biofilm by culture-dependent techniques. In total, and considering that several strains were capable of growing at different temperatures, 20 of these strains were isolated at 25 °C, while seven were isolated after incubation at 10 °C and eight were obtained by culturing at 4 °C (Fig. 2, Tables S1 and S2). At genus level, the microorganisms isolated at 25 °C belong to the genera *Acidovorax*, *Acinetobacter*, *Bacillus*, *Chryseobacterium*, *Delftia*, *Hydrogenophaga*, *Methylobacterium*, *Nocardia*, *Prolinoborus*, *Rhodococcus*, *Aspergillus*, *Neomicrosphaeropsis*, and *Penicillium*. At 10 °C, the isolates identified belonged to the genera *Bacillus*, *Peribacillus*, *Pseudomonas*, *Sphingomonas*, *Briansuttonomyces*, *Cadophora*, and *Vishniacozyma* while the genera *Flavobacterium*, *Pseudomonas*, *Sphingomonas*, *Cadophora*, *Filobasidium*, and *Vishniacozyma* were isolated at 4 °C (For more information about the species level of isolated microorganisms, see Tables S1 and S3). Some species were found in all the temperatures used for incubation. Strains belonging to the genera *Pseudomonas*, *Sphingomonas*, and *Cadophora* were isolated at all the aforementioned temperatures, while *Flavobacterium* sp. was identified at 4 and 25 °C, *Briansuttonomyces* sp. and *Bacillus* sp*.* were cultured at 10 and 25 °C, and *Vishniacozyma* sp. was isolated both at 4 and 10 °C. Those strains that were isolated on multiple media but whose 16S rRNA gene sequence was 100% identical were considered a single strain. Details on the media in which each strain was isolated can be found in Tables S1 and S2 for bacteria and fungi, respectively.

In general, the phylogenetically closest neighbours to our isolates, including non-culturable clones, displayed a wide ecological distribution, being present in cold and oligotrophic environments such as a freshwater lake in Antarctica, the Baltic Sea and freshwater recirculating aquaculture systems, a kart aquifer, the sediment of a Canadian glacier, a former coal-spoil site in the Arctic and in the water of a mesotrophic lake (Table S3). Overall, these environments share a similar pattern of low temperatures and oligotrophy. The closest environmental neighbours of the rest of the strains isolated from the ice machine drain pipe were associated to soil, human and plants (Table S3).

### Microbial community in the ice-machine biofilm analysed through NGS

#### The 16S rRNA and ITS amplicon sequencing

**Figure 3:**
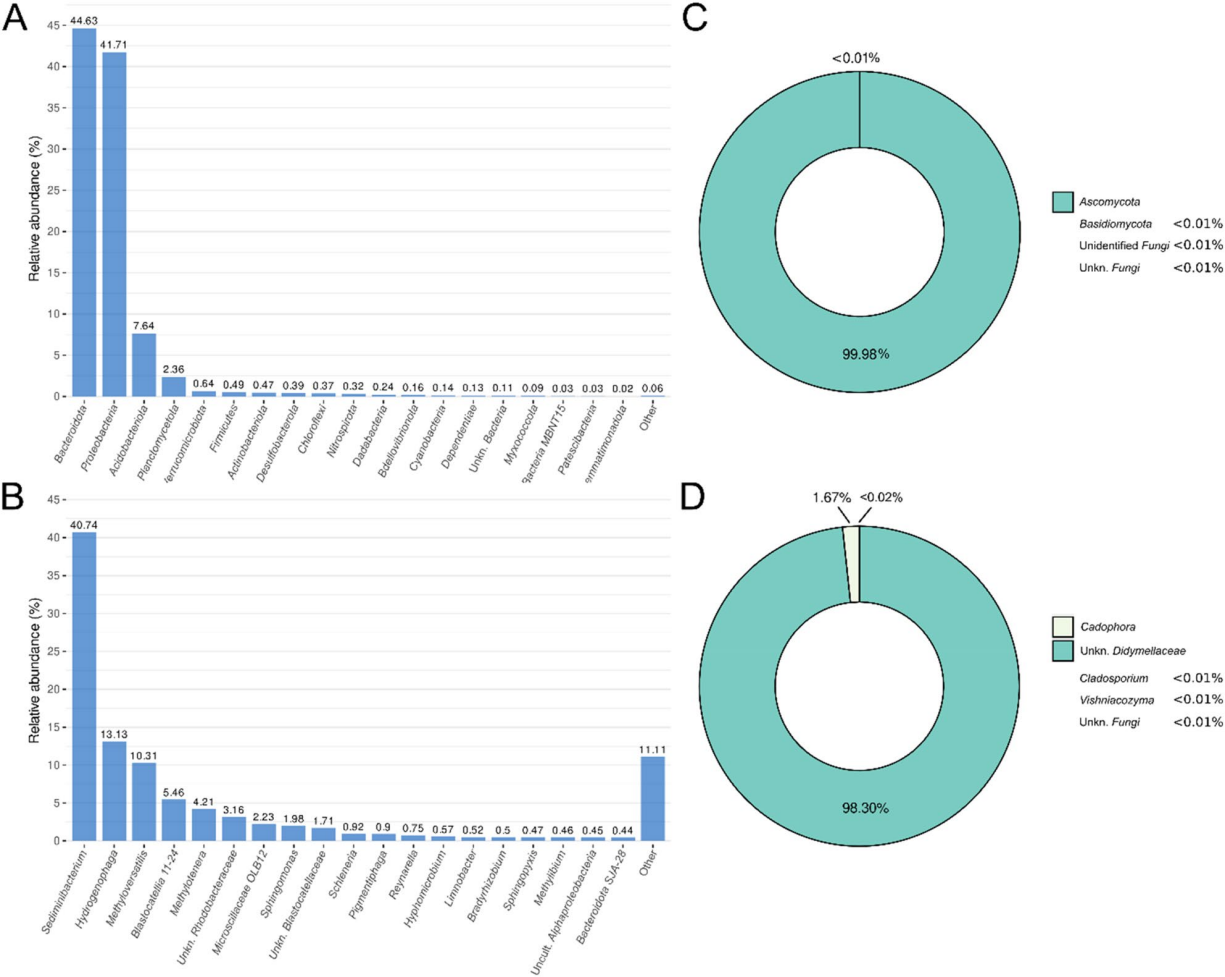
Taxonomic composition of the (**A**) bacterial community detected in the sample at phylum and (**B**) genus level; and the fungal community at (**C**) phylum level and (**D**) genus level.

In parallel to the culture-dependent study, the microbial community within the biofilm was analysed by 16S rRNA and ITS high-throughput sequencing. After trimming of reads, a total of 106,886 16S rRNA sequences and 149,989 ITS sequences were available for further analyses, corresponding to 1298 and 311 different ASVs, respectively. The entire diversity of this sample was captured, as the rarefaction curves were saturated (Fig. S1). *Bacteroidota* and *Proteobacteria* were the most abundant bacterial phyla in the sample, both with a relative abundance greater than 40%. Two other phyla, *Acidobacteriota* and *Planctomycetota*, showed an abundance greater than 10% (Fig. 3A). At the genus level, *Sediminibacterium* (*Bacteroidota* phylum) predominated with a relative abundance higher than 40%, followed by *Hydrogenophaga* and *Methyloversatilis* (both of which belong to the *Pseudomonadota* phylum) with a relative abundance exceeding 10% each (Fig. 3B). Of the 12 bacterial strains isolated, only two (*Peribacillus* sp. IM10-21 and *Pseudomonas* sp. strain IM4-26) were not detected by 16S rRNA gene analysis (Fig. S2A). Strains isolated from the genera *Hydrogenopahaga* and *Sphingomonas* were the most abundant, with a total relative abundance of more than 10%.

Results from ITS sequencing revealed that the fungal community was clearly dominated by the phylum *Ascomycota*, with almost 100% relative abundance (Fig. 3C). This high abundance is almost entirely explained by the presence of an unknown genus from the *Didymellaceae* family, whose relative abundance exceeded 98% (Fig. 3D). A BLAST analysis was performed to determine which members of this family had the highest sequence similarity to the most abundant ASVs in the biofilm. Three of these five ASVs (> 98% of total abundance; Table S4) aligned with an equal level of significance to different species from *Didymellaceae* family, such as *Didymella boeremae*, *Ascochyta phacae*, *Neomicrosphaeropsis italica* or *Briansuttonomyces eucalypti*, although the similarity was below 99.5% in all the cases; therefore, it was not possible to identify the specific *Didymellaceae* species in the sample. *Cadophora luteo-olivacea* and *Vishniacozyma victoriae* were also detected in low abundances after the BLAST analysis. Regarding the detection of the isolated fungal strains, four of the seven were among the ASVs found by ITS sequencing (those belonging to the genera *Cadophora*, *Vishniacozyma*, *Briansuttonomyces* and *Neomicrosphaeropsis*) (Fig. S2B). Among them, the most abundant in the sample was *Cadophora* sp. strain IM4-10 *(Ascomycota* phylum)*,* with more than 1.5% relative abundance, being the second most abundant fungal taxon.

### Microbial community of the ice machine biofilm via microbiome of other cold, oligotrophic natural and artificial environments

**Figure 4.**
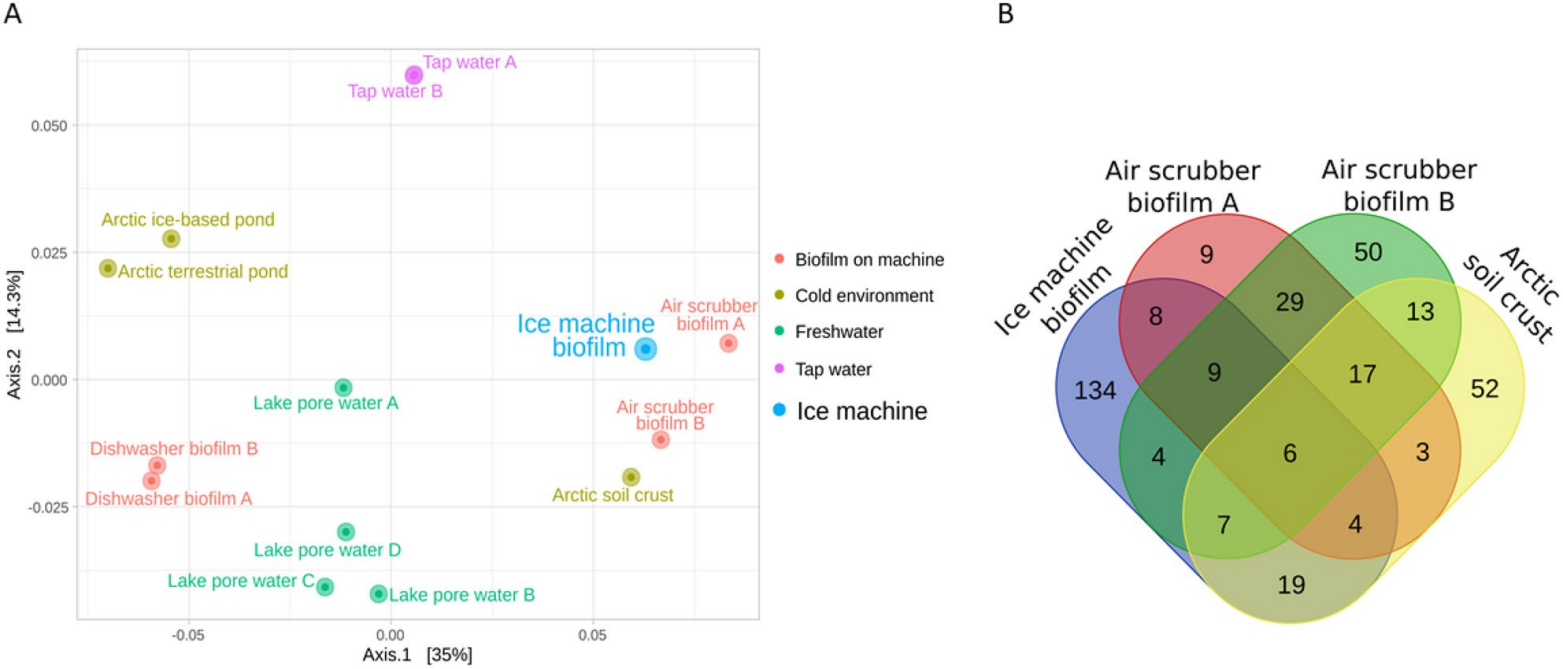
(**A**) PCoA of all the samples included in the beta diversity analysis. See Table S4 for additional information about the samples. (**B**) Venn diagram displaying the number of shared and unique genera among the ice machine biofilm and the biomes more closely related. Only genera with relative abundance greater than 0.01% were included in this analysis.

So as to assess the similarity of the ice machine biofilm to previously reported ones in terms of bacterial profile, a beta diversity analysis was carried out including this sample and others originating from cold, freshwater, tap water and artificial environments. The detailed list of the samples included in the analysis is available in Table S5. This meta-analysis was performed using the phylogeny-based weighted UniFrac metrics^13^, and revealed that the distance between this sample and all others was always higher than 0.08 (Table S6). This suggested that the selected samples presented marked differences in the bacterial composition. The Principal Coordinate Analysis (PCoA) plot (Fig. 4A) showed that the most similar samples to that of the ice machine biofilm were two biofilm samples coming from biological air scrubbers in a pig housing facility and an Arctic soil crust sample. In fact, despite the low similarity between samples, up to six genera identified through NGS (*Pseudoxanthomonas*, *Ferruginibacter*, unkn. *Comamonadaceae*, *Clostridium *sensu stricto* 1*, unkn. *Microbacteriaceae* and *Brevundimonas*) were shared by these four samples (Fig. 4B), while other 51 genera found in the ice machine microbiome were also found in at least one of the other samples. Finally, the alpha diversity analysis proved that the ice machine sample, despite showing a high number of observed ASVs, is relatively less diverse, that is, more homogeneous in its composition (Fig. S3).

#### Shotgun metagenomic sequencing

In order to complement the taxonomic information of the microbial community, shotgun metagenomics sequencing was also performed. Taxonomic analysis was performed on the filtered metagenomic raw reads (Fig. S4). More than 75% of the raw reads could not be classified because the corresponding taxa were not available in the database used. The family *Didymellaceae* was one of the taxa that was not available in this database at the time of analysis, so this taxon, which was predominant in the ITS analysis, could be included in this unclassified group in the metagenomic analysis. The next most abundant taxa were the genera *Methyloversatilis* and *Hydrogenophaga,* consistent with the 16S rRNA gene analysis performed, in which these two genera were the second and third most abundant. However, in this amplicon analysis, the most frequent genus was *Sediminibacterium,* which was not detected in the metagenomic sequences. With regard to the presence of the isolated strains, on the one hand, only three of the 18 isolated bacterial strains (those of the genera *Hydrogenophaga* and *Methylobacterium*, plus one of *Sphingomonas*) were detected by metagenomic sequencing. On the other hand, four of the seven fungal isolates (C*adophora, Briansuttonomyces, Neomicrosphaeropsis* and *Penicillium*) were identified by metagenomic sequencing. The *Penicillium* strain was the only one detected by metagenomic sequencing and not by amplicon sequencing.

In addition, shotgun reads were assembled, and binning was applied in order to recover metagenome-assembled genomes (MAGs) from the pool of contigs. A total of 47 and 52 bins were obtained with MaxBin2 and MetaBAT2, respectively. After running Das Tool, those non-redundant bins with the highest score were selected (Table S7). Following the defined standards by Bowers et al., eight of the isolated genomes could be considered as high-quality MAGs (completeness > 90% and contamination < 5%). Among them, four have a completeness > 99%. The other ten were medium-quality MAGs (completeness > 50% and contamination < 10%), where four of them have a completeness greater than 80% and contamination lower than 5%. Each MAG was taxonomically classified using GTDB-Tk. based on Average Nucleotide Identity (ANI) and/or placement in the class-level tree. Nine MAGs could be classified at the species level, while 5 others were defined at the genus level and 2 at the family level (Table S8). After attempting to obtain the 16S rRNA gene sequences of each MAG using Prokka, only MAGs 002, 015, 016, 13, 14, 20 and 38 could be retrieved. A BLAST was performed on each of these sequences using the 16S ribosomal RNA sequences (Bacteria and Archaea) from NCBI as a database. Only those performed with the 16S rRNA of MAGs 002, 14 and 13 gave significant results, although only for the first two did the results agree with those of GTDB-Tk at the genus level (data not shown). Furthermore, the coverage of each MAG was calculated with CoverM tool (Fig. S5). The MAG with the highest coverage was 002 (*Sediminibacterium* genus) with 11.9%, followed by 003 (*Hydrogenophaga* genus) and 11 (*Methyloversatilis* genus) with 8.5% and 6.3%, respectively. The remaining MAGs all had a coverage of less than 2%.

### Functional analysis of metagenomic data

**Figure 5:**
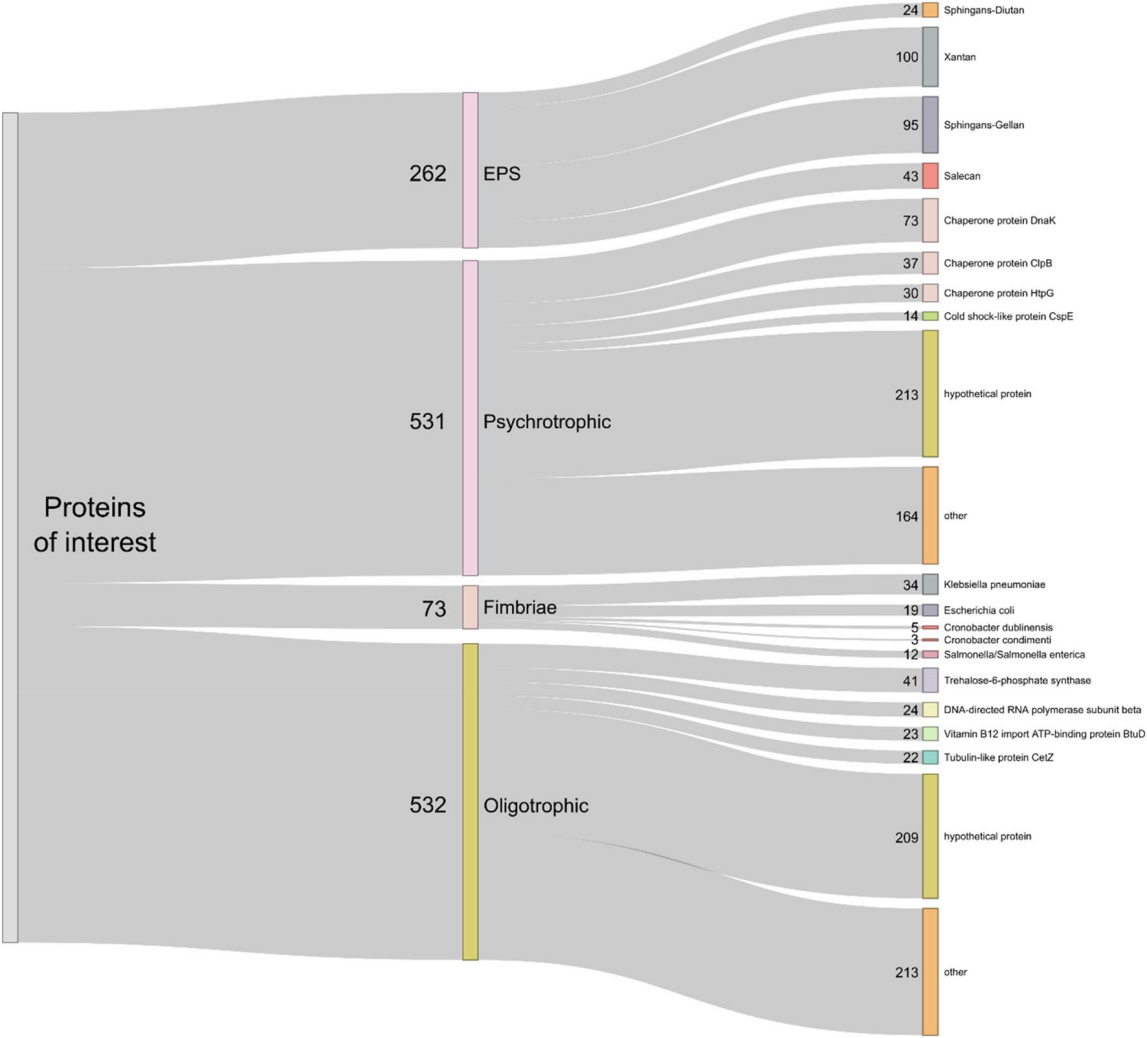
Map of all protein-coding genes of interest or homologs (bit score < 50) involved in EPS and fimbriae biosynthesis and tolerance to low temperature (psychrotrophic proteins) and oligotrophic conditions. See Table S9AD for additional information.

The functional profiling of the shotgun metagenome was carried out to identify genes and metabolic pathways involved in the adaptation of the microbial community to the cold oligotrophic conditions of the ice machine drainpipe. The result of the functional analysis confirmed the presence of proteins involved in the biosynthesis of extracellular polymers, such as salecan, xantan, succionglycan, hyaluronic acid (HA), alginate, colanic acid and sphingans -including gellan, welan, and diutan- (Fig. 5, Table S9A). Genes encoding proteins responsible for fimbriae biosynthesis such as K88 fimbriae (F4), fimbria biosynthesis transcriptional regulator, fimbriae usher protein, type 1 and 3 fimbriae were also identified (Fig. 5, Table S9B). The highest protein similarity scores were of 78.3 and 42.2% for EPSs and fimbriae biosynthesis, respectively. The bit score of the alignments for almost all proteins exceeded 50 bits.

Microorganisms adapt to survive cold environments through mechanisms such as biosynthesis of ice-binding protein (IBP), antifreeze proteins (AFPs), cold acclimation proteins (Caps) and cold shock proteins (Csps), among others^14–19^. To explore how they adapt to the cold temperature of ice machines, we searched for these proteins in our metagenome sequence dataset (Fig. 5, Table S9C). To identify oligotrophic metabolisms, we also searched for proteins involved in the biosynthesis of melanin, MELs, trehalose, mannitol, glycerol, beta-tubulin, and enzymes such as chitinases, succinate dehydrogenases, tryptophan synthetases, permeases, and six-hairpin glycosidases (Fig. 5, Table S9D). A significant number of genes could potentially contribute to tolerance in harsh environmental conditions such as low temperatures and oligotrophy. Among these, 262 genes showed homology to proteins involved in exopolysaccharide synthesis (alignment bit score > 50), 531 genes were homologous to psychrotrophic proteins, 532 genes were related to mechanisms to withstand oligotrophic conditions, and 73 genes had a potential function in fimbriae biosynthesis. Most of the EPS that could be expressed by the present genes corresponded to xanthan and gellan, and to a lesser extent to diutan and salecan. Among the psychrotrophic proteins, the large presence of genes potentially encoding chaperone (genes of the DnaK, ClpB, HtpG and ClpB types), as well as cold shock-like proteins of the CspE and CspA types, was noteworthy. Among the proteins that could be expressed by a larger number of genes to adapt to oligotrophic environments, the vitamin B12 import ATP-binding protein BtuD, the trehalose-6-phosphate synthase and the tubulin-like protein CetZ were highlighted. Most of the fimbria-forming proteins found were from *Klebsiella pneumoniae*, followed by *Escherichia coli* and *Salmonella* spp.*/Salmonella enterica*, while *Cronobacter dublinensis* and *C. condimenti* species had a lower prevalence.

**Figure 6:**
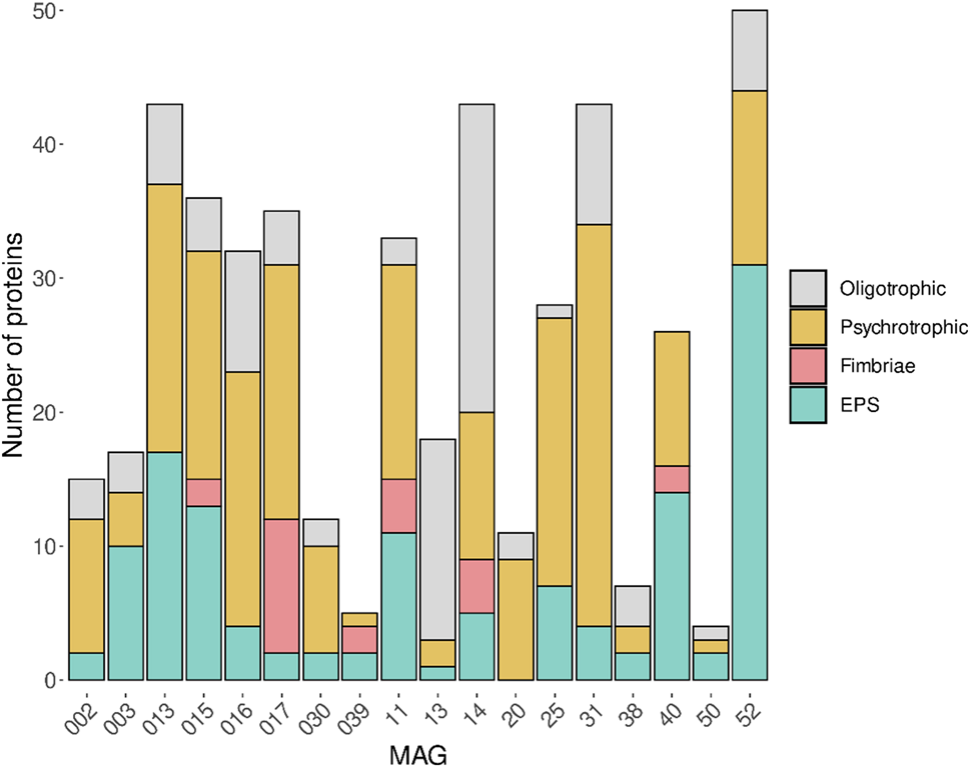
Number of different homologous proteins to known oligotrophic and psychrotrophic proteins and also those involved in EPS and fimbriae biosynthesis found in each MAG.

To assign which MAGs might be responsible for these metabolic functions, we searched the proteins of potential interest in each assembled MAG (Fig. 6). The result revealed that all MAGs have the genes involved in psychrophilic proteins biosynthesis, and also oligotrophic proteins presented in all MAGs except two; MAG 40 and 039. In particular, MAG 52 (*Sphingomonas* sp.) presented the most genes capable of expressing EPS, followed by others such as 013 (*Rhodobacteraceae JAAFHS01* sp.) and 40 (*Methyloversatilis discipulorum A*); among those with the greatest potential for fimbriae biosynthesis, 017 (*Pigmentiphaga sp009360345*) stood out; for the production of psychrotrophic proteins, the MAGs with the greatest potential were 31 (*Blastocatellia RBC074* sp.), 013, 016 (*Cyclobacteriaceae ELB16-189* sp.) and 017; and for the synthesis of proteins responsible for oligotrophic pathways, MAGs 13 (*Chryseoglobus sp002280615)* and 14 (*Mycobacterium gordonae*) were the most important ones.

We also searched for possible pathogenic microorganisms classified within risk group 2 or higher according to the Leibniz Institute DSMZ, which allowed us to detect several potential pathogens with very low abundance in the microbial community of the biofilm. In fact, the potential pathogenic microorganisms constituted less than 0.4% of the whole community (Fig. S6). From those, the most abundant pathogens were *Stenotrophomonas maltophila*, *Pseudomonas aeruginosa and Comamonas aquatica*.

## Discussion

In this work, we taxonomically and functionally characterised a very invasive microbial community that formed a biofilm in the drainpipe of a water pump coupled to a laboratory ice machine in our research institute. We aimed at providing the scientific community with clues about which microorganisms are able to colonise this anthropogenic environment -low temperature and oligotrophic- and the physiological mechanisms involved.

Previous studies have emphasized that ice machines can be, for example in hospitals, reservoirs of unsuspected opportunistic pathogens, which can cause infections. Ice machines have been found to be contaminated with opportunistic pathogens such as *Candida*, *Pseudomonas aeruginosa*, *Mycobacterium fortuitum*, carbapenem-resistant *Acinetobacter baumannii*, *Klebsiella pneumoniae* and *Stenotrophomonas maltophilia*^20–23^. However, there are no comprehensive reports describing the complete microbial communities in such a specific niche.

In our case, the microbial community, which formed a mucous, gummy biofilm within a few days, developed under cold oligotrophic conditions even after continuous chemical treatments in the drain pipe of various laboratory ice machines. Through high-throughput 16S rRNA and ITS amplicon sequencing, we unveiled that the bacterial community in the biofilm was dominated by members of the genera *Sediminibacterium*, *Hydrogenophaga* and *Methyloversatilis*. Members of these genera have a worldwide distribution, although some species were previously isolated from different aquatic environments such as hot springs^24^ and wastewater^25^ as well as in Antarctica^26,27^. *Hydrogenophaga* and *Methyloversatilis* have been reported to be psychrotrophic and psychrotolerant^28–30^. From an environmental perspective, strains closely related to the ones isolated from the ice machine such as *Acidovorax* sp., *Hydrogenophaga* sp., *Methylobacterium* sp., *Pseudomonas* sp., *Rhodococcus* sp., *Sphingomonas* sp., and *Filobasidium* sp. have previously been isolated in aquatic environments (Table S3) such as water purifiers, fresh water, pharmaceutical wastewater, cloud water, and a drinking water distribution system.

On the other hand, the fungal community was completely dominated by fungi from the family *Didymellaceae*, however, it was not possible to identify by ITS high throughput sequencing the main species in the sample. Interestingly, fungal strains of the species *Briansuttonomyces* sp. and *Cadophora* sp. were previously detected in different sites in the Arctic, and previous research revealed that the mycelia of *Briansuttonomyces* sp. and *Cadophora* sp. strains act as a network for the exchange of nutrients and more particularly elements such as carbon^31–33^. In general, fungi are present in artificial and semi-artificial environments such as drains in kitchens and bathrooms, sills beneath condensation-prone windows^34^, air-conditioning filters^35^, and dishwashers^36^ due to their ability to colonise nutrient-depleted environments also subjected to drastic temperature changes. Species of the *Didymellaceae* family have been identified as cold-adapted microorganisms in Antarctica^37^, and forests in the north of Poland^38^. In almost all these natural and artificial environments, nutritional resources are scarce, suggesting that the uniqueness of the ice machine microbiome could result from the effect of both selective pressures simultaneously, cold and nutrient shortage.

Further, we characterised the ice machine microbiome by comparing the results of the metataxonomic and metagenomics with the isolated bacteria and fungi. The taxonomic analysis could also capture the presence of most of the isolated microorganisms in the biofilm. The genera *Hydrogenopahaga* and *Sphingomonas* were the most abundant bacteria in the16S rRNA gene analysis; both were recovered by culture-dependent techniques. In addition, fungi from the genera *Cadophora*, *Vishniacozyma*, *Briansuttonomyces* and *Neomicrosphaeropsis* were also isolated by the culture-based method. A few of the isolated microorganisms were not detected in the amplicon sequencing analysis, suggesting that regardless of the favourable culturing conditions _which resulted in the isolation of those microbial cells_ they probably stood out in a very small fraction of the microbial community. In addition, as more than 75% of the raw reads of the shotgun metagenomic sequencing were not classified on the corresponding database, the metagenomic analysis confirmed the presence of only a few isolated bacteria and fungi. The results suggest that due to the limitations of each method, a combination of all techniques may provide more accurate and clear insight describing the microbial community of any environment.

It must be highlighted that the additional taxonomic analysis of the metagenome-assembled genomes that were recovered from the metagenomic analysis demonstrated that bacteria colonized the ice machine held a great taxonomic novelty, according to phylogenomic indices. Indeed, some MAGs could be only classified at the genus level. In fact, we studied the uniqueness of the ice machine microbial community in terms of beta diversity by comparing it with other microbiomes. This analysis revealed that biofilms in biological air scrubbers in a pig housing facility and Arctic soil crust were more similar to the ice machine biofilm than other samples from fresh and tap water included in the analysis. These closer samples shared up to six genera, some of which have been found in oligotrophic and cold environments, such as *Pseudoxanthomonas* and *Brevundimonas* in cold soils^39,40^ or *Ferruginibacter* in cold water^41^. Nevertheless, large differences were detected when comparing these microbiomes with the microbial community inhabiting the ice machine. For example, *Sediminibacterium*, the most abundant genus in the ice machine biofilm, was only detected in tap water and freshwater samples always under 2% abundance. As for *Hydrogenophaga* and *Methyloversatilis*, these genera were absent or appeared in minor abundances in all samples besides the ice machine one. Apparently, it is unusual to find biomes with such a high abundance of these genera, although its joint presence has been found in dental unit waterlines^42^, which are also enclosed areas with direct contact with water. In this later case, the relative abundances of these genera were substantially lower than in the ice machine.

Combining a set of media covering a wide range of nutritional requirements with three different incubation temperatures, we isolated 18 bacterial and seven fungal strains (Fig. 2). We were able to isolate mainly strains belonging to the phyla *Bacteroidota*, *Proteobacteria* and *Ascomycota*, although other non-abundant species belonging to the phylum *Actinomycetota* and *Bacillota* were also found. Despite the cold conditions, most of the isolated microorganisms were mesophilic strains able to survive at the low temperature of the ice machine. Similarly, the taxa with a higher relative abundance according to the NGS analysis (*Sediminibacterium*, *Methyloversatilis*, *Hydrogenophaga* and *Stagonosporopsis*) are mesophilic species. Although previous research reported that some isolated bacteria and fungi such as *Methylobacterium marchantiae* and *Vishniacozyma victoriae* can grow in a wide range of temperatures^26,43^, the biofilm could provide shelter and protection for some mesophilic microorganisms, that could remain metabolically inactive. Some environmental bacteria such as *Acidovorax temperans*, *Bacillus toyonensis, Delftia acidovorans, Hydrogenophaga palleronii, Methylobacterium marchantiae*, and *Pseudomonas lactis* tend to form dense biofilms^44–48^. Sporogenesis may also be playing a key role, as some species closely related to the strains present in the ice machine drain pipe such as *Bacillus toyonensis* and *Peribacillus simplex* were previously reported as spore-forming bacteria^48–50^.

The functional analysis carried out in this work confirmed the presence of proteins involved in cold adaptation mechanisms and oligotrophic metabolic pathways (Fig. 5, Table S9). To overcome the challenges of living in cold environments, cold-adapted microorganisms could develop unique mechanisms. These mechanisms include changes in the lipid profile of their membranes to increase fluidity^51,52^. This results in increased conformational flexibility and enzyme activity for important cellular processes such as transcription and translation^53^. They could also produce cryoprotectants such as cold shock protein (CSP)^54^, antifreeze protein (AFP)^55^ and even exopolysaccharide (EPS) to prevent freezing^56–58^.

Exopolysaccharide producing microorganisms have been isolated from diverse polar and cold environments^56,59–61^ including *Pseudomonas*^58^, *Pseudoalteromonas*^59^, *Flavobacterium*^62^ and *Colwellia*^60^ among others. Therefore, we hypothesise that the dense biofilm formed in the ice machine was one of the main adaptive strategies that the microorganisms developed to colonise this ecological niche. EPSs also are involved in microbial interactions, cell communications, and attachment to the surface^3^. Functional profiling provided us with a clear insight into the genes and the putative proteins presented in the microbial community of the ice machine biofilm. Thus, we analysed the functionality of the genes that are responsible for coding proteins involved in EPS and fimbriae biosynthesis. Previous studies suggest that the most reliable functional analysis for the shotgun sequences are homology-based methods versus available reference sequences in public databases^63^. However, homologous sequences do not always share a significant sequence similarity^64^. The most common reason is that the alignments of DNA sequences are less accurate than alignments of protein sequences, and due to this fact, for inferring protein homology from metagenome sequences, the bit score is a more accurate value than per cent identity^64^. Therefore, based on the bit scores of each alignment, the results of the functional analysis confirmed the presence of homologous proteins responsible for EPSs and fimbriae synthesis by the ice machine microbiome. Furthermore, checking the biosynthesis of EPSs in the assembled MAGs showed a higher number of the genes encoding EPSs _more than 30 protein encoding genes_ associated with the genus *Sphingomonas*, and to a lesser extent, to genera *Methyloversatilis*, and *Rhodobacteraceae.* This finding aligns with previous research, which highlighted the critical role of EPSs synthesized by *Sphingomonas* sp. for biofilm formation in the pipe material of the water distribution system, even in the presence of disinfectant reagents such as chlorine^65^.

Alternatively, the characterized microbiome could correspond to a pool of genomic variants of common mesophilic microorganisms, with specific, and yet to be explored, adaptations to cold, oligotrophic water. Intriguingly, the presence of the various psychrotolerant and psychrophilic proteins in all assembled MAGs suggests that the ice machine microbiome has already provided different adaptation mechanisms to cope with the environmental low temperature stress, even though a significant portion of the microbial community seems mesophilic. Future work could focus on analysing temperature growth ranges of all the strains to elucidate whether they are psychrophiles or low temperature-tolerant mesophiles.

Through the functional analysis of the metagenomic data, it was also possible to gather insights into the mechanisms associated with adaptations to oligotrophic environments. As described, a high number of genes that could code for the vitamin B12 import ATP-binding protein BtuD were detected. This protein is an ABC transporter that allows the assimilation of vitamin B12, which shares a good part of the subunits present in the iron assimilation and transport systems^65^. Another of the most detected proteins was the trehalose-6-phosphate synthase, a producer of trehalose, which has been linked to carbohydrate and energy storage functions and resistance to stress^66^. Different genes encoding chitinases were also found, which allow nitrogen and carbon to be obtained as a source of energy, nutrients and metabolic precursors^67^. In addition, a wide variety of genes encoding potential nutrient and metabolite transport proteins were detected, such as D-xylose transporter, magnesium-transporting ATPase, calcium-transporting ATPase, potassium-transporting ATPase, putative metabolite transport proteins CsbC and NicT, major myo-inositol transporter IolT and glycerol uptake facilitator protein. The functional analysis of the MAGs also uncovered, except two, all MAGs have a variety of genes encoding oligotrophic proteins, which allow microbial cells to be compatible in a nutrient deprivation environment such as the ice machine.

As stated before, the biofilm that colonized the ice machine drain pipe had been subjected to continued treatments with quaternary ammonium chloride. Quaternary ammonium compounds are cationic biocides widely used as disinfectants. These penetrate the bacterial cell membrane by electrostatic gravity and result in cell lysis. Previous works have demonstrated that positively charged quaternary ammonium ions are absorbed onto the surface of negatively charged *Sediminibacterium* and *Acinetobacter* species, altering cell wall permeability^66^. Microbial resistance to this family of compounds has been studied in common food contaminants such as *Staphylococci* and *Pseudomonas*, in which at least three resistance genes determine the resistance level of the strains^67^. Interestingly, studies have also found that the general level of resistance of pure cultures to quaternary ammonium compounds is low, whereas the EPS within biofilms increased surface hydrophilicity, for example, in *P. aeruginosa* and *S. aureus*, increasing the level of resistance relative to that of planktonic bacteria. Apart from the exopolysaccharide, it was also demonstrated that the three-dimensional structure of the biofilm cells (water channels) plays a key role in microbial resistance^68^. Despite the development of these adaptation mechanisms, a time-course metagenomic analysis revealed that microbial communities exposed to quaternary ammonium dramatically decreased in phylogenetic diversity and that the community adaptation occurred primarily via selective enrichment of quaternary ammonium degrading *Pseudomonas* strains, and secondarily via amino acid substitutions and horizontal transfer of a few genes such as genes encoding PAS/PAC sensor proteins and ring-hydroxylating dioxygenases^69^.

The potential pathogenic organisms stood at less than 0.4% of the whole microbial community. A low relative abundance of the health-threaten microorganisms revealed that the ice machine does not provide favourable conditions for colonization. Also, regardless of the NGS analysis, none of those pathogens was recovered by the culture-based methods. Indeed, as the favourable temperature for most of these pathogenic organisms is 30 to 37 ºC based on the information of the Leibniz Institute DSMZ for those microorganisms (https://www.dsmz.de), it was not surprising that the relative abundance of those pathogens is low compare to the environmental microorganisms in the biofilm community.

## Conclusions

In conclusion, in this case study, we describe the microbial composition of a biofilm that recurrently developed in a standard laboratory ice machine. Metataxonomic analysis indicates that the microbiome of the ice machine drain pipe biofilm is composed mainly of one or few fungal members closely related to the *Didymellaceae* family, and a relatively diverse bacterial community, dominated at the genus level by *Sediminibacterium*, *Hydrogenophaga* and *Methyloversatilis*. Moreover, alpha and beta diversity analysis demonstrate that the microbiome is different to those of other cold and humid environments (although it shares some important genera). At a higher taxonomic resolution (the one that MAGs provide), we have found that bacteria detected in the ice machine correspond to a number of potential new taxa, which we have not been able to culture to date.

The functional analysis of the shotgun reads also uncovered the presence of key proteins involved in biofilm development such as EPS biosynthesis and fimbriae production, cold adaptation mechanisms and oligotrophic metabolisms^51–60,62^. The functional analysis of the assembled MAGs could also be studied to gather more comprehensive information on the adaptation mechanisms and the power of living in a biofilm under harsh conditions.

Although there are many factors that could influence the microbiome in ice machines, including the working conditions, the influx water or the manufacturing materials among others, in this particular case, the microbial community that formed the biofilm may have been greatly shaped by the use of a chemical treatment. Whereas it is not possible to establish the microbiome reported in this study as a representative or standard microbiome of laboratory ice machines, this work demonstrates that the daily use of man-made devices can be a surprisingly fruitful source of new microbial taxa.

## Experimental procedures

### Sample collection

Two daily-use standard laboratory ice machines (ITV Ice Makers; Spain; S/N: 17634427) installed in two different floors at the Institute for Integrative Systems Biology (I2SysBio; 39.5167° N, 0.4231° W) with an approximate room temperature of 23 °C started to clog in the summer of 2019. Since then, a treatment with Green Pantabs (Highside Chemicals Inc.; USA) consisting of quaternary ammonium chloride started to be applied every two weeks. However, machine failure due to clogging continued to occur. In August 2021, a large piece of a pale, light brown, gummy biofilm was harvested from the out-flux pipe of one of the laboratory ice machines (ITV Ice Makers; Spain; S/N: 17634427) and stored in a 50 ml tube at 4 °C. The temperature and pH of the ice machine water reservoir and the sample were recorded: 5 °C and 8.2 ± 0.1, respectively.

### Media and solutions

Eight different media and Phosphate Buffer Saline (PBS) solution were used for strain isolation from the ice machine biofilm. These were selected to cover a wide range of nutritional requirements in order to promote the growth of diverse bacterial and fungal species (For more information about the compositions of media and solution, see Table S10).

### Strain isolation and identification

Immediately after collecting the sample from the out-flux pipe of the laboratory ice machine, a fraction of the biofilm was transferred to a 1.5 ml tube and physically smashed by using an adapted plastic pestle and then vortexing to obtain a homogenized mixture. Serial dilutions were prepared using PBS and plated on eight different media (R2A, LB, TSA, MA, YM, CA, BHI, Efm2). The plates were incubated for at least 15 days at 25 °C, 10 °C and 4 °C under aerobic conditions. Single colonies were periodically isolated and re-streaked on new plates until pure strains were obtained. These were cryo-conserved in 15% glycerol at − 80 °C.

For taxonomic identification, a colony of pure culture was resuspended in 100 µL of sterile MilliQ-water and subjected to three consecutive heat shock cycles (99 °C; 3 min and 4 °C; 3 min). This mixture was used to amplify either the 16S rRNA gene or the ITS region. The 16S rRNA PCR was performed using the *Taq* PCR Master Mix Kit (Qiagen, Germany), and the following PCR cycle: initial denaturation at 94 °C for 3 min; 30 cycles of amplification (30 s at 94 °C, 30 s at 50 °C, 1 min 30 s at 72 °C); and 10 min of extension at 72 °C, using the universal primers that amplify the 16S of ribosomal RNA gene 8F (5′-AGA GTT TGA TCC TGG CTC AG-3′) and 1492R (5′-CGG TTA CCT TGT TAC GAC TT-3′)^70^. To amplify the ITS2 hypervariable region of the fungal nuclear ribosomal DNA (rDNA), PCRs were carried out using the primers 3F (5′-GCA TCG ATG AAG AAC GCA GC-3′) and 4R (5′-TCC TCC GCT TAT TGA TAT GC-3′)^71^ and the following PCR cycle: initial denaturation at 95 °C for 3 min; 30 cycles of amplification (30 s at 94 °C, 30 s at 53 °C, 30 s at 72 °C); and 10 min of extension at 72 °C. The amplification of the targeted regions was confirmed by gel electrophoresis (1.2% agarose in 0.5X Tris–Borate-EDT buffer), and the PCR products were purified using a mixture of isopropanol and 3 M potassium acetate (10:1 v/v). Amplicons were sequenced by Sanger sequencing (Eurofins Genomics; Germany). All the sequences were manually trimmed before comparing them against the EzBioCloud () and NCBI online databases (). EzBioCloud was used to taxonomically identify the closest type strains for the bacterial isolates, whereas for the fungal strains NCBI was used applying the following constrains: Nucleotide Blast; rRNA/ITS databases, 16S ribosomal RNA sequences or Internal Transcribed spacer region (ITS), limit to sequences from type material. Additionally, in order to gather information regarding the ecology of the isolated strains, the trimmed sequences were compared against the NCBI standard nucleotide collection (nr/nt).

### Metagenomic DNA isolation

Another fraction of the biofilm was transferred to a 1.5 ml tube and physically smashed and homogenized by vortexing, as mentioned in the culture-based method, to break down the gummy sticky biomass before DNA extraction. Then, the homogenized mixture was treated using the DNeasy PowerSoil kit (Qiagen, Germany) following the manufacturer's instructions. The DNA was eluted in 20 µL of MilliQ water pre-warmed at 65 °C to facilitate DNA recovery. The extracted DNA was quantified using Qubit dsDNA HS Assay kit (Qubit 2.0 Fluorometer, Q32866).

### Metataxonomic analysis

In order to study the bacterial and fungal communities present in the biofilm, the extracted metagenomic DNA was used to amplify the hypervariable region V3-V4 of the 16S ribosomal RNA gene and the ITS2 hypervariable region of the fungal nuclear ribosomal DNA (rDNA). The conserved regions V3 and V4 (459 bp) of the 16S rRNA gene were amplified using the following forward and reverse primers: 5′-TCG TCG GCA GCG TCA GAT GTG TAT AAG AGA CAG CCT ACG GGN GGC WGC AG 3′ and 5′-GTC TCG TGG GCT CGG AGA TGT GTA TAA GAG ACA GGA CTA CHV GGG TAT CTA ATC C-3′, and the following PCR cycle: initial denaturation at 95 °C for 3 min; 25 cycles of amplification (30 s at 95 °C, 30 s at 55 °C, 30 s at 72 °C); and 5 min of extension at 72 °C^72^. The DNA amplicon libraries of the ITS2 region were generated using the primers ITS3-F_KYO2 (18S SSU 2029–2046) and ITS4_KYO1 (5.8 2390–2409)^73^, and a PCR cycle consisting of: initial denaturation at 95 °C for 3 min, 28 cycles of amplification (30 s at 95 °C, 30 s at 58 °C, 30 s at 72 °C), and 5 min of a final extension step at 72 ºC. In both cases, the amplification was carried out using the KAPA HiFi HotStart ReadyMix PCR kit (KK2602). The 16S rRNA and ITS2 amplicons were mixed with Illumina sequencing barcoded adaptors (Nextera XT index kit v2, FC-131-2001), and libraries were normalized and merged. The pools with indexed amplicons were loaded onto the MiSeq reagent cartridge v3 (MS-102-3003) and spiked with 10% PhiX control to improve the sequencing quality, that was finally conducted using paired-ends on an Illumina MiSeq platform (2 × 300 bp) in the Foundation for the Promotion of Health and Biomedical Research of the Valencian Community (Fisabio) (Valencia, Spain).

The raw Illumina sequences were loaded into Qiime2 (v. 2021.2.0)^74^. The quality of the sequences was checked using the plugin Demux and the Qiime2-integrated DADA2 pipeline was used for trimming and joining the sequences, removing chimeras and detecting amplicon sequence variants (ASVs) (> 99.9% of similarity). The taxonomy of each sequence variant was determined via the classify-Sklearn module from the feature-classifier plugin, employing SILVA (v. 138)^75^ and UNITE (v. 8.2)^76^ as reference databases for 16S rRNA and ITS2 taxonomic assignment, respectively. Additionally, the most abundant ASVs resulting from ITS sequencing were classified with BLAST against the NCBI’s ITS database. Results were analysed with the phyloseq R package (v. 1.30.0)^77^.

### Shotgun metagenomics analysis

The extracted DNA was prepared for whole genome sequencing by sonication. The obtained DNA fragments were polished, A-tailed, ligated with the following adaptors 5′-AGA TCG GAA GAG CGT CGT GTA GGG AAA GAG TGT AGA TCT CGG TGG TCG CCG TAT CAT T-3′ and 5′-GAT CGG AAG AGC ACA CGT CTG AAC TCC AGT CAC GGA TGA CTA TCT CGT ATG CCG TCT TCT GCT TG-3′ and amplified by PCR. The PCR products were purified with the AMPure XP system for library preparation, and the size distribution of the libraries was checked by Agilent 2100 Bioanalyzer (Agilent Technologies, CA, USA) and quantified by real-time PCR. Sequencing was conducted using Illumina NovaSeq 6000 platform (2 × 150 bp).

Adaptors were trimmed from raw reads with Cutadapt (v. 3.4)^78^, and quality filtering was performed with BBDuk, from the BBTools package (Bushnell B., https://sourceforge.net/projects/bbtools/ updated January 2, 2018). Quality-checked (QC) reads were mapped against the *Homo sapiens* reference genome (GRCh38.p13, https://www.ncbi.nlm.nih.gov/assembly/GCF_000001405.39) using Bowtie2 (v. 2.3.5.1)^79^ to detect and filter any human-related reads. The quality of the reads was checked before and after the filtering using FastQC (v. 0.11.9) (). For taxonomic analysis of these filtered reads, the Kraken2 tool^80^ was used with the PlusPF-16 database (including archaea, bacteria, viruses, plasmids, human, protozoa, fungi and UniVec).

QC reads were then assembled with MEGAHIT (v. 1.2.9)^80^, and assembly statistics were obtained with QUAST (v. 5.0.2)^81^. The filtered reads were mapped to the assembled contigs using Bowtie2, and a sorted BAM file was obtained with SAMtools (v. 1.13)^82^. Contig abundance statistics were obtained using the jgi summarize bam contig depth script from MetaBAT pipeline^83^. Then, binning was carried out by MetaBAT (v. 2.12.1)^83^, and MaxBin (v. 2.2.7) (https://www.sourceforge.net/projects/maxbin2/) using the default parameters. Metagenome-assembled genomes (MAGs) were selected by using DAS Tool (v. 1.1.3)^84^, which compares the quality of the MAGs recovered by the binning tools and chooses the best ones. The quality of those MAGs was further evaluated with CheckM (v. 1.1.3)^85^.

The taxonomic annotation of the MAGs was conducted by employing the Genome Taxonomy Database Toolkit (GTDB-Tk, v2.1.1)^86^, with GTDB R07-RS207 as the reference dataset. Coverage of MAGs was calculated by read mapping with CoverM (v. 0.5.0) (Woodcroft, 2007; CoverM. https://github.com/wwood/CoverM). Finally, prokka (v. 1.14.6)^87^ was used for annotating the MAGs and detect its 16S rRNA sequence.

Functional profiling was also used to analyse different biological activities within the microbial community. One of the objectives of the present study was to search for proteins involved in EPS synthesis, fimbriae production, cold adaptation mechanisms and also oligotrophic metabolic pathways in the ice machine biofilm. Therefore, starting from a bibliographic search of proteins already described with some of these functions, the search for these proteins and/or homologous proteins was carried out among the genes present. For this purpose, Prodigal v. 2.6.3 was used to predict the presence of genes in the assembly, as well as to obtain the amino acid sequences derived from them. From these, it was determined with BlastP v. 2.11.0+ which genes showed complete or partial homology with some of the proteins already described as involved in EPS, fimbriae production, cold shock mechanisms and oligotrophic metabolic pathways. Based on the previous research, homologous proteins were considered to have a bit score ≥ 50^64^.

In addition, a search for possible pathogenic organisms in the biofilm sample was carried out in order to detect any pathogens classified as the risk group 2 or higher in the Leibniz Institute DSMZ (German Collection of Microorganisms and Cell Cultures GmbH), which is based on Directive 2000/54/EC of the European Parliament and of the Council of 18 September 2000 on the protection of workers from risks related to exposure to biological agents at work environments.

### Scanning electron microscopy (SEM)

The structural and morphological characteristics of the ice machine biofilm were analysed through SEM. Small fragments of the biofilm were fixed by immersion into Karnovsky's Fixative (2% paraformaldehyde, 2.5% glutaraldehyde in 0.1 M sodium phosphate buffer; pH 7.4) overnight, then gently washed with sterile deionized water and a series of ethanol solutions (30, 50, 70, 90, and 100% ethanol absolute; PanReac, AppliChem). Samples were incubated for 48 h in the desiccator prior to embedding them in resin using carbon tape. Sputtering was done using Gold/Palladium (Au/Pd) particles and samples were examined under the Field Emission Scanning Electron Microscope (FE-SEM) Hitachi S4800 (SCSIE, University of Valencia).

### Supplementary Information

Supplementary Information.

Supplementary Table 9.

## Data Availability

All raw reads of amplicon and metagenomics analyses can be found on NCBI's Sequence Read Archive (SRA) under the Bioproject Accession PRJNA781810. In addition, the codes utilized for data analysis and figure generation have been archived on GitHub at the following URL: https://github.com/danieltorsil/IceMachine. Please contact the corresponding author at alba.iglesias@uv.es to request data from this study.
