# Supplementary material for: A laboratory ice machine as a cold oligotrophic artificial microbial niche for biodiscovery
Source: Sci Rep. 2023 Dec 12;13:22089. doi: 10.1038/s41598-023-49017-0 (PMC10716499; doi:10.1038/s41598-023-49017-0)
Supplement: Supplementary file 1 — Supplementary Information. [file 41598_2023_49017_MOESM1_ESM.docx]

**A laboratory ice machine as a cold oligotrophic artificial microbial niche for biodiscovery**

Leila Satari^1^, Daniel Torrent^2^, Asier Ortega-Legarreta^2^, Adriel Latorre-Pérez^2^, Javier Pascual^2^, Manuel Porcar^1,2^ and Alba Iglesias^1^

**Supplementary Tables**

**Supplementary table 1.** Bacterial strains isolated from the biofilm formed on the ice machine drain pipe isolated at 4, 10, and 25 ºC with the top closest neighbour (EzBioCloud; [https://www.ezbiocloud.net](https://www.ezbiocloud.net/)). CA (Columbia Blood Agar), BHI (Blood Heart Infusion), LB (Lysogenic Broth), YM (Yeast Mold), TSA (Tryptic Soy Agar), R2A (Reasoner's 2A agar), Efm2, MA (Marine Agar).

| **Strain** | **Closest type strain** | **Similarity**  **(%)** | **Isolation Media** | **4ºC** | **10ºC** | **25ºC** | **Accession Number** |
| --- | --- | --- | --- | --- | --- | --- | --- |
| **IM-2b** | *Acidovorax temperans* CCUG 11779^T^ | 99.40 | R2A, BHI | - | - | + | OL634869 |
| **IM-27** | *Prolinoborus fasciculus* CIP 103579^T^ | 100.00 | R2A, TSA, CA | - | - | + | OL634876 |
| **IM10-5** | *Bacillus toyonensis* BCT-7112^T^ | 100.00 | LB, YM | - | + | + | OL634885 |
| **IM-16b** | *Chryseobacterium hispalense* DSM 25574^T^ | 100.00 | TSA, CA | - | - | + | OL634875 |
| **IM-12** | *Delftia acidovorans* 2167^T^ | 100.00 | TSA, YM | - | - | + | OL634872 |
| **IM-15** | *Flavobacterium quisquiliarum* EA-12^T^ | 98.97 | R2A, TSA, CA, LB, YM | + | - | + | OL634873 |
| **IM-32a** | *Hydrogenophaga palleronii* NBRC 102513^T^ | 99.90 | TSA | - | - | + | OL634878 |
| **IM-41** | *Methylobacterium marchantiae* JT1^T^ | 99.90 | YM | - | - | + | OL634880 |
| **IM-16a** | *Nocardia asteroides* NBRC 15531^T^ | 99.12 | CBI, BHI | - | - | + | OL634874 |
| **IM-31** | *Nocardia rhizosphaerihabitans* KLBMP S0039^T^ | 99.27 | TSA, LB, CA | - | - | + | OL634877 |
| **IM10-21** | *Peribacillus simplex* NBRC 15720^T^ | 100.00 | LB | - | + | - | OL634886 |
| **IM4-26** | *Pseudomonas lactis* DSM 29167^T^ | 100.00 | R2A, TSA, CA, YM, BHI, LB | + | + | + | OL634883 |
| **IM-9** | *Prolinoborus fasciculus* CIP 103579^T^ | 100.00 | YM | - | - | + | OL634871 |
| **IM-38** | *Rhodococcus cerastii* C5^T^ | 99.70 | TSA | - | - | + | OL634879 |
| **IM-42** | *Rhodococcus fascians* C5^T^ | 99.53 | MA | - | - | + | OL634881 |
| **IM4-31** | *Sphingomonas aerolata* NW12^T^ | 99.63 | R2A, TSA, CA, LB | + | + | + | OL634884 |
| **IM4-19** | *Sphingomonas faeni* MA-olki^T^ | 99.91 | R2A, LB | + | - | - | OL634882 |
| **IM4-X** | *Sphingomonas faeni* MA-olki^T^ | 98.96 | R2A, LB | + | - | - | OL638313 |

**Supplementary table 2.** Fungal strains isolated from the biofilm, their accession number on NCBI, and similarities to the closest type strains (BLAST, NCBI rRNA/ITS databases, 16S ribosomal RNA sequences or Internal Transcribed spacer region (ITS), limit to sequences from type material; <https://blast.ncbi.nlm.nih.gov/Blast.cgi>). CA (Columbia Blood Agar), BHI (Blood Heart Infusion), LB (Lysogenic Broth), YM (Yeast Mold), TSA (Tryptic Soy Agar), R2A (Reasoner's 2A agar), Efm2, MA (Marine Agar).

| **Isolated fungal strains** | **The closest type strain** | **Similarity**  **(%)** | **Isolation Media** | **4ºC** | **10ºC** | **25ºC** | **Accession Number** |
| --- | --- | --- | --- | --- | --- | --- | --- |
| **IM-6a** | *Aspergillus austroafricanus* | 99.68 | YM | - | - | + | OL634947 |
| **IM10-13** | *Briansuttonomyces eucalypti* | 99.33 | BHI, YM, MA | - | + | + | OL634952 |
| **IM4-10** | *Cadophora luteo-olivacea* | 99.32 | R2A, TSA, CA, BHI, YM, Efm2 | + | + | + | OL634949 |
| **IM4-11** | *Filobasidium magnum* | 99.74 | LB | + | - | - | OL634950 |
| **IM-17b** | *Neomicrosphaeropsis italica* | 99.30 | TSA | - | - | + | OL634948 |
| **IM-5** | *Penicillium citrinum* | 99.68 | YM | - | - | + | OL634946 |
| **IM10-8** | *Vishniacozyma victoriae* | 99.30 | CA, YM | + | + | - | OL634951 |

**Supplementary table 3.** Strains isolated from the biofilm formed on the ice machine drain pipe and their similarities to the closest environmental strains/clones (BLAST, NCBI standard nucleotide collection (nr/nt); <https://blast.ncbi.nlm.nih.gov/Blast.cgi>). The isolation source of the environmental strains/clones indicates in the table. NA (not available).

| **Isolated strain** | **The closest environmental strains/clones (Accession Number)** | **Similarity**  **(%)** | **Environmental strain/clone (Isolation source)** | **Reference** |
| --- | --- | --- | --- | --- |
| IM-2b | *Acidovorax delafieldii* strain 179 (EU730925.1)  Uncultured bacterium clone d_65_AG_Prop_242 (JN090788.1)  Uncultured bacterium clone ncd362e12c1 (HM313242.1) | 100.00  99.88  99.88 | Water Purifiers in Gunsan Area  Aerobic granules cultivated on propionate in a laboratory sequencing batch reactor  Skin, antecubital fossa | Seo *et al*., 2009 ^1^  Benardini *et al*., 2005 ^2^  Kong *et al., 2012* ^3^ |
| IM-27 | *Acinetobacter lwoffii* strain FDAARGOS_557 (CP054803.1)  *Acinetobacter lwoffii* strain FDAARGOS_551 (CP054822.1)  *Acinetobacter* sp. strain H111 (MH671625.1) | 100  100  100 | Clinical isolate  Clinical isolate  Pine tree | FDA database, 2013 ^4^  FDA database, 2013 ^4^  Ponpandian *et al.*,2019 ^5^ |
| IM10-5 | *Bacillus toyonensis* strain WS1-2 (MT605501.1)  *Bacillus thuringiensis* strain DT-3 (MT588729.1) | 100.00  100.00 | NA  NA | _  _ |
| IM-16b | *Bacterium* strain BS1114 (MK824302.1)  *Bacterium* strain BS1096 (MK824284.1)  Uncultured bacterium clone A15 (MG744647.1)  *Chryseobacterium hispalense* strain FPBSKK1 (KU605691.1) | 100.00  100.00  100.00 | Tomato rhizosphere  Gut content of a freshwater fish species  Sweet pepper | Gu, S. *et al.,* 2020 ^6^  Dutta *et al.*, 2015 ^7^  Füstös *et al.*, 2017 ^8^ |
| IM-12 | *Delftia* sp. S17 (HE662648.1)  *Delftia acidovorans* strain DS (MN173541.1)  Uncultured bacterium clone LS-TB-4 (MH052675.1) | 100.00  99.91  99.91 | Slope soil from south gate of Nanjing Agricultural University  Mud from Dead Sea resort in Jordan  Rhizosphere soil | _  Ivanov *et al.*, 2018 ^9^  _ |
| IM-15 | *Flavobacterium quisquiliarum* strain ME2 (MN326710.1)  Uncultured bacterium clone nbw1182e06c1 (GQ079897.1)  *Flavobacterium* sp. PVR09 (KP072754.1) | 99.38  99.38  99.38 | Ginseng soil  The human skin  Plant | _  Grice *et al*., 2009 ^10^  _ |
| IM-32a | Uncultured beta proteobacterium clone O-5-26 (KF827217.1)  Uncultured bacterium (FR853427.1)  *Hydrogenophaga* sp. Gsoil 1545 (AB271047.1) | 100  100  100 | Biofilm from fresh water  NA  Soil of a ginseng field | Zhang *et al.*, 2014 ^11^  _  Im w. & Lee S. 2005 ^12^ |
| IM-41 | *Methylobacterium* sp. strain A3K057 (MN989081.1)  *Methylobacterium* marchantiae strain R100B/2 (KR811206.1)  *Methylobacterium* sp. PK29_S1 (JF274801.1) | 100.00  100.00  100.00 | Compound leaves of Fraxinus excelsior  Freshwater Lake Radok, East Antarctica  Olive-mill wastewater | Ulrich *et al.*, 2020 ^13^  Karlov *et al.*, 2017 ^14^  Tsiamis *et al.*, 2012 ^15^ |
| IM-16a | *Nocardia* sp. JSM 147630 (KR817792.1)  *Nocardia* sp. JSM 147631 (KR817793.1) | 100.00  100.00 | Forest soil in Hunan, China | _ |
| IM-31 | *Nocardia neocaledoniensis* strain NRK66 (MK841332.1)  *Nocardia asteroides* strain APN00049 (KC262094.1)  *Nocardia neocaledoniensis* strain DSM 44717 (NR_118204.1) | 99.79  99.79  99.79 | Bronchoalveolar lavage (BAL)  Sputum  NA | _  _  Conville *et al.*, 2010 ^16^ |
| IM10-21 | *Bacillus* sp. strain HBUM207125 (MT598008.1)  *Peribacillus simplex* strain ILQ109 (MN826155.1)  *Peribacillus simplex* strain CsMF-18 (MT415154.1) | 100.00  100.00  100.00 | NA  Rhizosphere of Chenopodium quinoa  Stalactite | _  Chumpitaz *et al.*, 2020 ^17^  _ |
| IM4-26 | *Pseudomonas gessardii* strain ST3SE (MN069032.1)  *Pseudomonas libanensis* strain FA67 (MT271892.1)  *Pseudomonas* sp. strain 3JPC (MT084587.1) | 100.00  100.00  100.00 | Soil  Pharmaceutical wastewater  Garlic | _  _  _ |
| IM-9 | *Acinetobacter lwoffii* strain FDAARGOS_557 (CP054803.1)  *Acinetobacter lwoffii* strain FDAARGOS_551 (CP054822.1)  *Prolinoborus* sp. strain B9 (MT576572.1) | 100.00  100.00  100.00 | Clinical isolate  Clinical isolate  Skin lesions of *Salmo trutta fario* | Sproer *et al.*, 2021 ^18^  Sproer *et al.*, 2021 ^18^  _ |
| IM-38 | *Rhodococcus* sp. 2Dben2 (JX177726.1)  *Rhodococcus* sp. strain 140616R8 (MK036524.1)  *Rhodococcus* sp. I_GA_K_5_3 (FJ267568.1) | 99.80  99.80  99.90 | Baltic Sea surface water  Freshwater RAS systems  Kitchen air | Vedler *et al.*, 2013 ^19^  _  _ |
| IM-42 | *Rhodococcus* sp. PDD-41b-2 (JF706542.1)  *Rhodococcus* sp. strain Raf1(MT012177.1)  *Rhodococcus* sp. TPH-S8 (KP091903.1) | 99.91  99.81  99.81 | Cloud water  NA  Total petroleum hydrocarbon contaminated soil | Vaïtilingom *et al.*, 2012 ^20^  Dhaouadi *et al.*, 2020 ^21^  _ |
| IM4-31 | *Sphingomonas* sp. strain C2-1(MT279969.1)  *Sphingomonas* sp. strain A3K041 (MN989146.1)  *Sphingomonas* sp. strain ICMP 22266 (MH392690.1) | 100.00  100.00  100.00 | A simulated drinking water distribution system  Compound leaves of Fraxinus excelsior  Water | Zhu, Z. *et al.*, 2020 ^22^  _  _ |
| IM4-19 | *Sphingomonas* sp. strain D3P057 (MN989175.1)  *Sphingomonas* sp. strain PDD-32b-57 (MN686253.1)  *Sphingomonas* sp. G16 (JQ977017.1) | 99.91  99.91  99.91 | Compound leaves of Fraxinus excelsior  NA  Bulk soil | Ulrich *et al.*, 2020 ^13^  _  _ |
| IM4-X* | Uncultured bacterium clone EDW07B005_19 (HM066598.1)  Uncultured bacterium clone 9_B09 (FN421797.1)  Uncultured bacterium clone 4_E01 (FN421750.1) | 99.56  99.19  99.12 | A karst aquifer, Texas state well  Phyllosphere of soybean  Phyllosphere of soybean | Gray *et al.*, 2013 ^23^  Deimotte *et al.*, 2009 ^24^  Deimotte *et al.*, 2009 ^24^ |
| IM-6a | *Aspergillus tabacinus* strain DUCC5721 (MT582761.1)  *Aspergillus versicolor* strain DUCC5711 (MT582751.1)  *Aspergillus versicolor* isolate R47 (MT420642.1) | 99.68  99.68  99.68 | An Asthma patient houses  An Asthma patient houses  Barber shops in informal settlements | _  _  _ |
| IM10-13 | *Phoma herbarum* isolate GR5-3-20-2 (LC515071.1)  *Phoma herbarum* strain JN0398 (MK359683.1)  *Phoma herbarum* strain IS179 (LC085217.1) | 100.00  100.00  100.00 | Sediment of Walker glacier, Canadian High Arctic  Plant  Moss | Tsuji *et al.*, 2019 ^25^  Nelson *et al.*, 2019 ^26^  Hirose *et al.*, 2017 ^27^ |
| IM4-10 | *Cadophora malorum* isolate IVA-283 (MN833360.1) *Cadophora malorum* strain IS090 (LC085207.1)*Cadophora fastigiata* strain F-30 (MF077223.1) | 100.00  100.00  100.00 | Former coal-spoil sites in Arctic  Moss  Deep root plant rhizosphere | Iliushin *et al.*, 2022 ^28^  Hirose *et al.*, 2017 ^27^  *Zhang et al.*, 2017 ^29^ |
| IM4-11 | *Filobasidium magnum* (MT635292.1)  *Filobasidium* sp. isolate FBFY28 (MK186942.1)  *Filobasidium magnum* strain TY4 (MK226288.1) | 99.74  99.74  99.74 | Mesotrophic lake water  Plant  Wheat | Adam C. & Magdalena Ś., 2022 ^30^  _  Solanki *et al.*, 2019 ^31^ |
| IM-17b | *Phoma herbarum*strain N-20Ps-2-2-1 (KJ191690.1)  *Phoma herbarum* strain N-18Sm-2-2-1 (KJ191684.1)  *Phoma herbarum* GR5-3-20-2 (LC515071.1) | 100.00  100.00  100.00 | NA  NA  Sediment | _  _  Tsuji *et al.*, 2019 ^25^ |
| IM-5 | *Penicillium citrinum* strain DUCC5728 (MT582768.1)  *Penicillium citrinum* strain EE104-F2 (MT560285.1)  *Cladosporium* sp. isolate BC15-5 (MT383120.1) | 99.68  99.68  99.68 | An Asthma Patients House  Closed Habitat  NA | _  Malli Mohan *et al.*, 2020 ^32^  _ |
| IM10-8 | *Vishniacozyma carnescens* strain AD191(MN922485.1)  *Tremellomycetes* sp. strain V07 (MF614974.1)  *Vishniacozyma carnescens* strain AY805 (MG250423.1) | 100.00  100.00  100.00 | Endophytic yeasts of durum wheat  In-growth mesh bags in barren ground  Inner surface of termite's tapetumsamples from Namib Desert | Alfonzo *et al.*, 2021 ^33^  Falbesoner *et al.*, 2018 ^34^  _ |

*Possible new species

_ Unpublished data (information about the isolation source of each microorganism is available at; <https://blast.ncbi.nlm.nih.gov/Blast.cgi>)

**Supplementary table 4.** DNA sequences of 5 most abundant ASVs.

| **ASV** | **Relative abundance (%)** | **DNA sequence (5’-3’)** |
| --- | --- | --- |
| 28aafbae6847410c759150195aeaa4ef | 96.24 | AATGCGATAAGTAGTGTGAATTGCAGAATTCAGTGAATCATCGAATCTTTGAACGCACATTGCGCCCCTTGGTATTCCATGGGGCATGCCTGTTCGAGCGTCATTTGTACCTTCAAGCATTGCTTGGTGTTGGGTGTTTGTCTCGCCTTTGCGTGTAGACTCGCCTTAAAACAATTGGCAGCCGGCGTATTGATTTCGGAGCGCAGTACATCTCGCGCTTTGCACTCATAACGACGACGTCCAAAAGTACATTTTAACACTCTTGACCTCGGATCAGGTAGGGATACCCGCTGAACTT |
| e3cbd9d4f2411ae392c1169679e9b9d4 | 2.05 | AATGCGATAAGTAGTGTAAATTGCAGAATTCAGTGAATCATCGAATCTTTGAACGCACATTGCGCCCCTTGGTATTCCATGGGGCATGCCTGTTCGAGCGTCATTTGTACCTTCAAGCATTGCTTGGTGTTGGGTGTTTGTCTCGCCTTTGCGTGTAGACTCGCCTTAAAACAATTGGCAGCCGGCGTATTGATTTCGGAGCGCAGTACATCTCGCGCTTTGCACTCATAACGACGACGTCCAAAAGTACATTTTAACACTCTTGACCTCGGATCAGGTAGGGATACCCGCTGAACTT |
| 95c62bf7b2c516651322dd8be08c4ff6 | 1.68 | AATGCGATAAGTAATGTGAATTGCAGAATTCAGTGAATCATCGAATCTTTGAACGCACATTGCGCCCTCTGGTATTCCGGGGGGCATGCCTGTTCGAGCGTCATTATAACCACTCAAGCTCTCGCTTGGTATTGGGGTTCGCGGTTCCGCGGCCCCTAAAATCAGTGGCGGTGCCTGTCGGCTCTACGCGTAGTAATACTCCTCGCGTCTGGGTCCGGTAGGTCTACTTGCCAGCAACCCCCAATTTTTACAGGTTGACCTCGGATCAGGTAGGGATACCCGCTGAACTT |
| 0d741b9c5dc1974201603223bb3c73ec | 0.01 | CCTTCTCTCCTTCTTTTTCCTTTCCTCCTTCCTTGAATCATCGAATCTTTGAACGCACATTGCGCCCCTTGGTATTCCATGGGGCATGCCTGTTCGAGCGTCATTTGTACCTTCAAGCATTGCTTGGTGTTGGGTGTTTGTCTCGCCTTTGCGTGTAGACTCGCCTTAAAACAATTGGCAGCCGGCGTATTGATTTCGGAGCGCAGTACATCTCGCGCTTTGCACTCATAACGACGACGTCCAAAAGTACATTTTAACACTCTTGACCTCGGATCAGGTAGGGATACCCGCTGAACTT |
| 73cde2de5d622cd81a2e5d090782bfe6 | 0.01 | AATGCGATAAGTAATGTGAATTGCAGAATTCAGTGAATCATCGAATCTTTGAACGCACCTTGCGCCCTTTGGTATTCCGAAGGGCATGCCTGTTTGAGTGTCATGAAACCTCACCCCACTTGGGTTTTTGCCTGAGCGGTGGTGTATTGGGTGTTGCCTTGCCAAAGGCTCGCCTTAAAAACATAAGCACCTTGGATGTAATACGTTTCATCCTTCTGGGTGGCTGATAACCCCACATATTCATGATCTGGCCTCAAATCAGGTAGGGCTACCCGCTGAACTT |

**Supplementary table 5.** Samples included in the beta diversity analysis. All samples come from studies in which the microbiome was determined by 16S rRNA V3-4 region sequencing.

| **Biome** | **Sample Name** | **Description** | **Reference** | **Run accession** |
| --- | --- | --- | --- | --- |
| Cold environment | Arctic terrestrial pond | Arctic-Terrestrial Pond in tundra | Kleinteich *et al*., 2017 ^35^ | ERR2204459 |
|  | Arctic ice-based pond | Arctic - Ice-based Pond | Kleinteich *et al*., 2017 ^35^ | ERR2204492 |
|  | Arctic soil crust | Arctic - Soil crust | Kleinteich *et al*., 2017 ^35^ | ERR2204489 |
| Freshwater | Lake pore water A | France - Lake Grangent pore water | Keshri *et al*., 2018 ^36^ | SRR5749817 |
|  | Lake pore water B | France - Lake Aydat pore water | Keshri *et al*., 2018 ^36^ | SRR5749812 |
|  | Lake pore water C | France - Lake Pavin pore water | Keshri *et al*., 2018 ^36^ | SRR5749804 |
|  | Lake pore water D | Austria - Lake Neusiedl pore water | von Hoyningen-Huene *et al*., 2019 ^37^ | SRR8266734 |
| Tap water | Tap water A | France - Tap water in Paris | Perrin *et al*., 2019 ^38^ | ERR2611918 |
|  | Tap water B | France - Tap water in Paris | Perrin *et al*., 2019 ^38^ | ERR2611911 |
| Biofilm on machines | Dishwasher biofilm A | Slovenia - biofilm from dishwasher | Raghupathi *et al*., 2018 ^39^ | SRR3343800 |
|  | Dishwasher biofilm B | Denmark - biofilm from dishwasher | Raghupathi *et al*., 2018 ^39^ | SRR3343755 |
|  | Air scrubber biofilm C | Belgium - biofilm from air scrubber from a pig housing facility | Van der Heyden *et al*., 2019 ^40^ | SRR7477781 |
|  | Air scrubber biofilm D | Belgium - biofilm from air scrubber from a pig housing facility | Van der Heyden *et al*., 2019 ^40^ | SRR7477802 |
| Ice machine | Ice machine biofilm | Biofilm clogging water pumps in ice machines | This study | PRJNA782825 |

**Supplementary table 6.** Unweighted UniFrac dissimilarity matrix.

|  | Dishwasher biofilm A | Dishwasher biofilm B | Air scrubber biofilm A | Air scrubber biofilm B | Arctic terrestrial pond | Arctic ice-based pond | Arctic soil crust | Lake pore water A | Lake pore water B | Lake pore water C | Lake pore water D | Tap water A | Tap water B |
| --- | --- | --- | --- | --- | --- | --- | --- | --- | --- | --- | --- | --- | --- |
| Dishwasher biofilm B | 0.014 |  |  |  |  |  |  |  |  |  |  |  |  |
| Air scrubber biofilm A | 0.160 | 0.158 |  |  |  |  |  |  |  |  |  |  |  |
| Air scrubber biofilm B | 0.143 | 0.141 | 0.059 |  |  |  |  |  |  |  |  |  |  |
| Arctic terrestrial pond | 0.120 | 0.118 | 0.171 | 0.157 |  |  |  |  |  |  |  |  |  |
| Arctic ice-based pond | 0.117 | 0.116 | 0.154 | 0.148 | 0.076 |  |  |  |  |  |  |  |  |
| Arctic soil crust | 0.141 | 0.139 | 0.083 | 0.067 | 0.155 | 0.148 |  |  |  |  |  |  |  |
| Lake pore water A | 0.115 | 0.113 | 0.130 | 0.118 | 0.115 | 0.120 | 0.119 |  |  |  |  |  |  |
| Lake pore water B | 0.108 | 0.107 | 0.121 | 0.119 | 0.136 | 0.131 | 0.123 | 0.100 |  |  |  |  |  |
| Lake pore water C | 0.126 | 0.125 | 0.149 | 0.132 | 0.139 | 0.132 | 0.127 | 0.110 | 0.124 |  |  |  |  |
| Lake pore water D | 0.113 | 0.112 | 0.131 | 0.119 | 0.127 | 0.125 | 0.127 | 0.101 | 0.065 | 0.120 |  |  |  |
| Tap water A | 0.119 | 0.115 | 0.115 | 0.119 | 0.136 | 0.125 | 0.125 | 0.103 | 0.121 | 0.134 | 0.113 |  |  |
| Tap water B | 0.119 | 0.115 | 0.116 | 0.118 | 0.135 | 0.125 | 0.124 | 0.102 | 0.121 | 0.133 | 0.113 | 0.003 |  |
| **Ice machine biofilm** | **0.150** | **0.148** | **0.081** | **0.087** | **0.158** | **0.145** | **0.093** | **0.120** | **0.114** | **0.142** | **0.122** | **0.114** | **0.114** |

**Supplementary table 7.** Summary statistics of the reconstructed metagenome-assembled genomes (MAGs), and selected by DAS Tool. The completeness and contamination were estimated with CheckM and the number of CDS (protein coding sequence) with Prokka. G + C, guanine-cytosine content. High-quality MAGs in grey (Bowers *et al*., 2017) ^41^.

| **MAG** | **Contigs** | **Genome Length**  **(Mb)** | **N50** | **Completeness**  **(%)** | **Contamination (%)** | **G + C (%)** | **CDS** | **Genome Accession Number** |
| --- | --- | --- | --- | --- | --- | --- | --- | --- |
| 002 | 69 | 3.5 | 122230 | 99.01 | 1.97 | 36.32 | 3091 | GCA_021155285.1 |
| 003 | 110 | 4.5 | 72755 | 74.23 | 3.56 | 67.05 | 4338 | GCA_021155305.1 |
| 11 | 206 | 3.4 | 24610 | 89.2 | 2.19 | 65.72 | 3176 | GCA_021155265.1 |
| 13 | 273 | 2.3 | 10164 | 85.58 | 1.52 | 70.41 | 2241 | GCA_021155185.1 |
| 013 | 52 | 3.7 | 153103 | 99.7 | 1.44 | 64.66 | 3657 | GCA_021155205.1 |
| 14 | 685 | 6.4 | 12260 | 91.88 | 1.71 | 66.51 | 6132 | GCA_021154995.1 |
| 015 | 67 | 3.0 | 98499 | 99.57 | 0.05 | 42.40 | 2821 | GCA_021155245.1 |
| 016 | 53 | 1.1 | 872541 | 100 | 3.99 | 40.15 | 4910 | GCA_021155195.1 |
| 017 | 156 | 6.1 | 70907 | 95.73 | 1.22 | 67.36 | 5638 | GCA_021155165.1 |
| 20 | 549 | 2.7 | 5077 | 76.96 | 3.59 | 55.57 | 2716 | GCA_021155145.1 |
| 25 | 1226 | 6.1 | 5241 | 66.53 | 4.38 | 70.72 | 5667 | GCA_021155115.1 |
| 030 | 999 | 2.8 | 3558 | 83.17 | 3.29 | 52.66 | 2959 | GCA_021155105.1 |
| 31 | 112 | 9.4 | 166518 | 90.54 | 3.42 | 56.44 | 7046 | GCA_021155085.1 |
| 38 | 22 | 3.4 | 224598 | 98.33 | 0.95 | 32.19 | 2881 | GCA_021155045.1 |
| 039 | 944 | 1.7 | 1922 | 59.41 | 2.28 | 55.77 | 1798 | GCA_021155065.1 |
| 40 | 93 | 3.5 | 69131 | 76.76 | 0.41 | 66.71 | 3169 | GCA_021155025.1 |
| 50 | 184 | 3.6 | 30378 | 83.67 | 1.17 | 65.64 | 3412 | GCA_021154985.1 |
| 52 | 501 | 3.8 | 9404 | 84.61 | 1.82 | 66.02 | 3537 | GCA_021154945.1 |

**Supplementary table 8.** Taxonomic affiliation and novelty of each metagenome-assembled genome (MAG). The taxonomic affiliation of each MAG was assessed using GTDB-Tk, which can be based on Average Nucleotide Identity (ANI) and/or placement in the class-level tree. High-quality MAGs in grey (Bowers *et al*., 2017) ^41^.

| **MAG** | **Classification** | **Classification based on** | **ANI (%) with closest assembly** |
| --- | --- | --- | --- |
| 002 | d__Bacteria;p__Bacteroidota;c__Bacteroidia;o__Chitinophagales;f__Chitinophagaceae;g__Sediminibacterium;s__Sediminibacterium sp017996795 | topological placement and ANI | 95.47 (GCA_017996795.1) |
| 003 | d__Bacteria;p__Proteobacteria;c__Gammaproteobacteria;o__Burkholderiales;f__Burkholderiaceae;g__Hydrogenophaga;s__Hydrogenophaga sp018993155 | topological placement and ANI | 97.15 (GCA_018993155.1) |
| 013 | d__Bacteria;p__Proteobacteria;c__Alphaproteobacteria;o__Rhodobacterales;f__Rhodobacteraceae;g__JAAFHS01;s__ | placement in class-level tree | 78.41 (GCA_013298485.1) |
| 015 | d__Bacteria;p__Proteobacteria;c__Gammaproteobacteria;o__Burkholderiales;f__Methylophilaceae;g__Methylotenera;s__Methylotenera mobilis_B | topological placement and ANI | 98.02 (GCF_000384255.1) |
| 016 | d__Bacteria;p__Bacteroidota;c__Bacteroidia;o__Cytophagales;f__Cyclobacteriaceae;g__ELB16-189;s__ | placement in class-level tree | 83.5 (GCA_016787755.1) |
| 017 | d__Bacteria;p__Proteobacteria;c__Gammaproteobacteria;o__Burkholderiales;f__Burkholderiaceae;g__Pigmentiphaga;s__Pigmentiphaga sp009360345 | topological placement and ANI | 99.32 (GCA_009360345.1) |
| 030 | d__Bacteria;p__Proteobacteria;c__Gammaproteobacteria;o__Burkholderiales;f__Burkholderiaceae;g__Limnobacter;s__Limnobacter sp002954425 | topological placement and ANI | 97.08 (GCF_013004065.1) |
| 039 | d__Bacteria;p__Desulfobacterota_D;c__UBA1144;o__UBA2774;f__UBA2774;g__;s__ | placement in class-level tree | - |
| 11 | d__Bacteria;p__Proteobacteria;c__Gammaproteobacteria;o__Burkholderiales;f__Rhodocyclaceae;g__Methyloversatilis;s__Methyloversatilis sp016791045 | topological placement and ANI | 98.25 (GCA_016791045.1) |
| 13 | d__Bacteria;p__Actinobacteriota;c__Actinomycetia;o__Actinomycetales;f__Microbacteriaceae;g__Chryseoglobus;s__Chryseoglobus sp002280615 | ANI | 95.2 (GCA_002280615.1) |
| 14 | d__Bacteria;p__Actinobacteriota;c__Actinomycetia;o__Mycobacteriales;f__Mycobacteriaceae;g__Mycobacterium;s__Mycobacterium gordonae | topological placement and ANI | 98.84 (GCF_002101675.1) |
| 20 | d__Bacteria;p__Acidobacteriota;c__Blastocatellia;o__Pyrinomonadales;f__Pyrinomonadaceae;g__OLB17;s__ | placement in class-level tree | 77.61 (GCA_902826795.1) |
| 25 | d__Bacteria;p__Desulfobacterota_B;c__Binatia;o__UBA12015;f__UBA12015;g__VGTK01;s__ | placement in class-level tree | 82.68 (GCA_016874715.1) |
| 31 | d__Bacteria;p__Acidobacteriota;c__Blastocatellia;o__RBC074;f__RBC074;g__RBC074;s__ | placement in class-level tree | - |
| 38 | d__Bacteria;p__Bacteroidota;c__Bacteroidia;o__AKYH767;f__2-12-FULL-35-15;g__;s__ | placement in class-level tree | - |
| 40 | d__Bacteria;p__Proteobacteria;c__Gammaproteobacteria;o__Burkholderiales;f__Rhodocyclaceae;g__Methyloversatilis;s__Methyloversatilis discipulorum_A | topological placement and ANI | 96.34 (GCF_000385375.1) |
| 50 | d__Bacteria;p__Proteobacteria;c__Gammaproteobacteria;o__Burkholderiales;f__Burkholderiaceae;g__Hydrogenophaga;s__ | placement in class-level tree | - |
| 52 | d__Bacteria;p__Proteobacteria;c__Alphaproteobacteria;o__Sphingomonadales;f__Sphingomonadaceae;g__Sphingomonas;s__ | placement in class-level tree | - |

**Supplementary table 9.** (Please check **Supplementary Excel**): The functional profiling of the shotgun metagenomes revealed the presence of (**A**) proteins involved in the biosynthesis of extracellular polymers, (**B**) Genes encoding proteins responsible for fimbriae biosynthesis (**C**) proteins associated with the cold adaptation mechanisms, and (**D**) proteins involved in oligotrophic metabolic pathways. The presence of homologous proteins responsible for EPSs and fimbriae synthesis as well as proteins associated with the cold adaptation mechanisms and oligotrophic metabolic pathways by the ice machine microbiome were confirmed by the alignments bit scores ≥ 50 (Pearson, 2019) ^42^.

**Supplementary table 10.** The compositions and pH of the media and solution used to isolate microorganisms in this study.

| **Medium** | **Medium compositions (g/L)** | **pH** | **Produced by** | **Reference** |
| --- | --- | --- | --- | --- |
| Reasoner's 2 Agar medium **(R2A)** | Peptone;1, Yeast Extract; 0.5, Dextrose/Glucose; 0.5, Soluble Starch; 0.5, Na_2_HPO_4_; 0.3, MgSO_4_.7H_2_O; 0.05, Sodium Pyruvate; 0.3, Agar; 15.0 | 7.2 (±0.2) | Lab-made medium | Ciric *et al*, 2022 ^43^ |
| Lysogenic Broth medium **(LB)** | Tryptone; 10, Yeast Extract; 5, NaCl; 10, Agar; 15 | 7.2 (±0.2) | Lab-made medium | Sagen *et al*, 2023 ^44^ |
| Trypticase Soy Broth medium **(TSA)** | Tryptone; 15.0, Soya Peptone; 5.0, NaCl; 5.0, Agar; 15.0 | 7.2 (±0.2) | Lab-made medium | Satari *et al*, 2020 ^45^ |
| Marine Agar medium **(MA)** | H_3_BO_3_; 0.022, NH_4_NO_3_; 0.0016, CaCl_2_; 1.8, SrCl_2_; 0.034, Yeast Extract; 1.0, Iron Citrate; 0.1, MgCl_2_; 8.8, Bile Salt n°3; 5.0, KBr, 0.55, NaCl; 19.4, NaF; 0.0024, NaHCO_3_; 0.16, Na_2_HPO_4_; 0.008, Na_2_SiO_3_; 0,004, Na_2_SO_4_; 3.24. Agar; 15.0 | 7.6 (±0.2) | PanReac Química SLU; Spain | Satari *et al*, 2022 ^46^ |
| Yeast Mold Agar medium **(YM)** | Yeast Extract; 3.0, Malt Extract; 3.0, Dextrose; 10.0, Peptone Soybean; 4.0, Agar;15.0 | 6.2 (±0.2) | Lab-made medium | Sohlberg *et al*, 2022 ^47^ |
| Columbia Agar medium **(CA)** | Special Peptone; 23.0, Starch;1.0, NaCl; 5.0, Agar;10.0 | 7.3 (±0.2) | Oxoid Ltd., UK | Feng *et al*, 2023 ^48^ |
| Brain Heart Infusion Agar medium **(BHI)** | Brain Heart Infusion Solids; 17.5, Peptone; 10.0, Glucose; 2.0, NaCl; 5.0, Na_2_HPO_4_; 2.5, Agar; 15.0 | 7.4 (±0.2) | VWR International bvba, EC | ^49^ |
| **M17** Broth medium | Tryptone; 2.5, Meat Peptone; 2.5, Soya Peptone; 5.0, Yeast Extract; 2.5, Meat Extract; 5.0, Sodium glycerophosphate; 19.0, MgSO_4_; 0.25, Ascorbic acid; 0.5 and Lactose; 5.0 | 7.4 (±0.2) | SIGMA ALDRICH-MERCK; Spain | Vasmara *et al*, 2021 ^50^ |
| **Efm2** | Glucose; 10.0, Saccharose; 3.0, Agar;15.0 adding to M17 Broth medium ; 42 | 7.4 (±0.2) | Lab-made medium | _ |
| Phosphate Buffer Saline **(PBS)** solution | NaCl; 8.0, KCl; 2.0, Na_2_HPO_4_; 1.44, KH_2_PO_4_; 0.24 | 7.4 | Lab-made medium | Satari *et al*, 2020 ^45^ |

**Supplementary Figures**


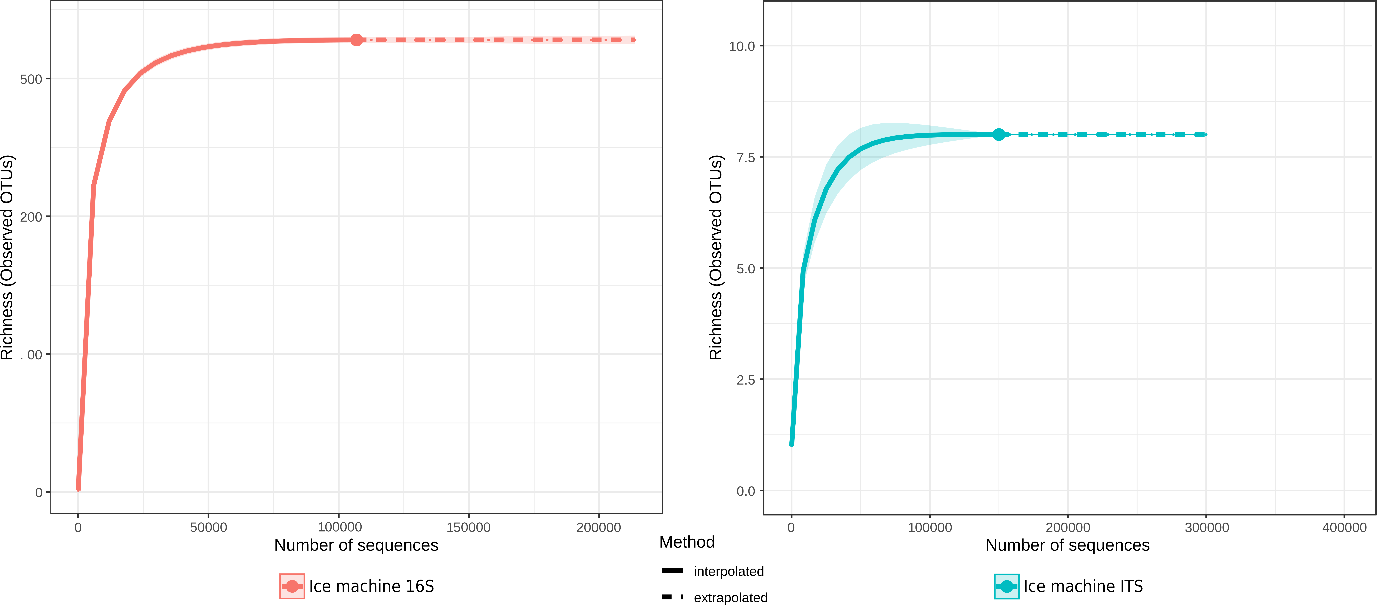


**Supplementary Figure 1.** Rarefaction curves for the 16S ribosomal RNA and ITS2 analyses in the ice machine biofilm sample**.**


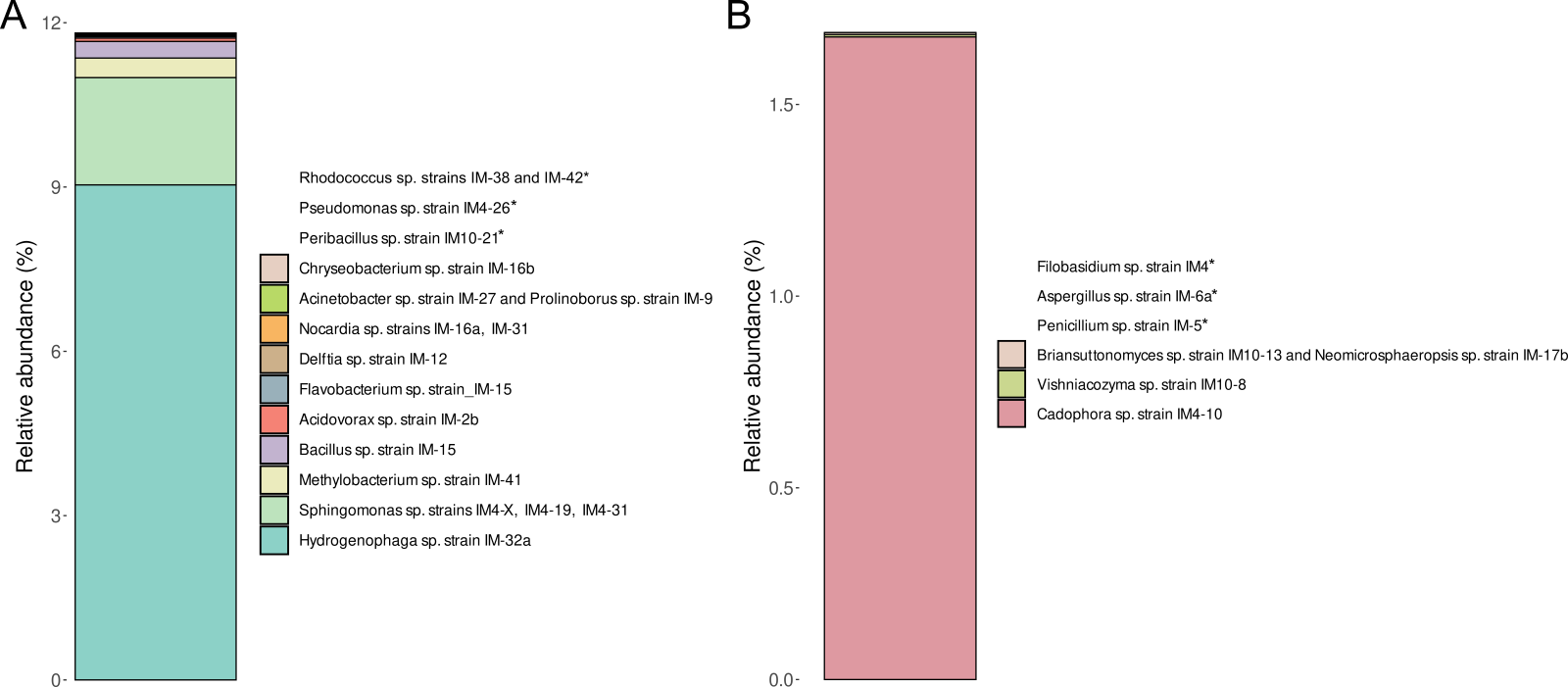


**Supplementary Figure 2**. Relative abundances of all isolated strains in 16S rRNA (A) and ITS2 (B) sequencing. Strains marked with an asterisk were not detected in the analyses.


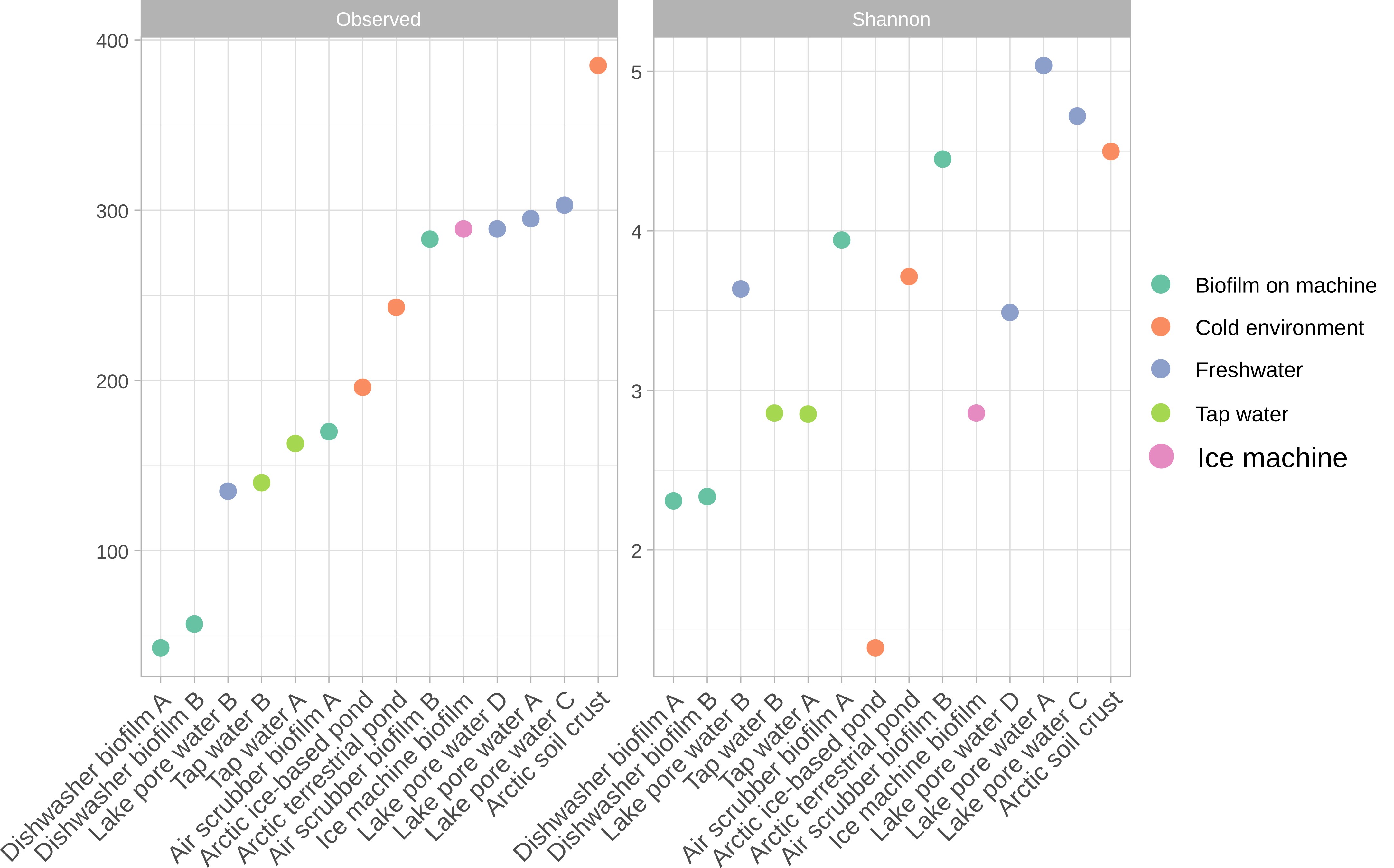


**Supplementary Figure 3.** Alpha diversity of all samples included in beta diversity analysis, with number of observed ASVs (total number of different microorganisms found in the sample) and Shannon index (total number of different microorganisms detected corrected by their relative abundance).


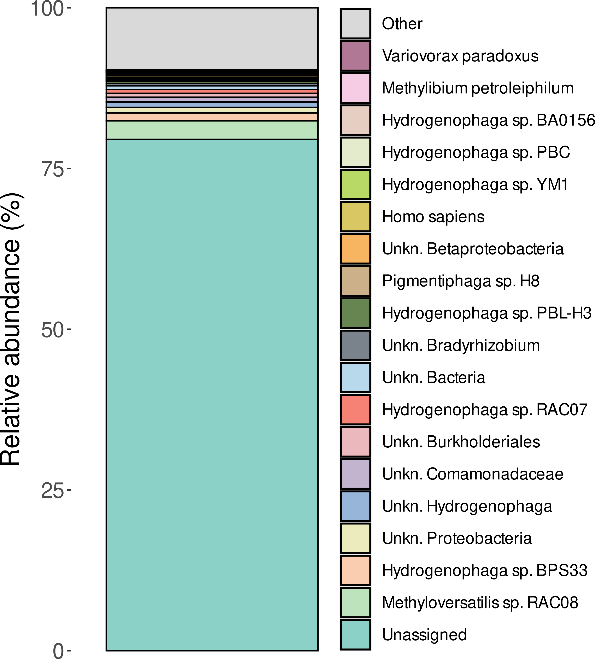


**Supplementary Figure 4.** Relative abundances of major taxa found in the biofilm using metagenomic sequencing data.


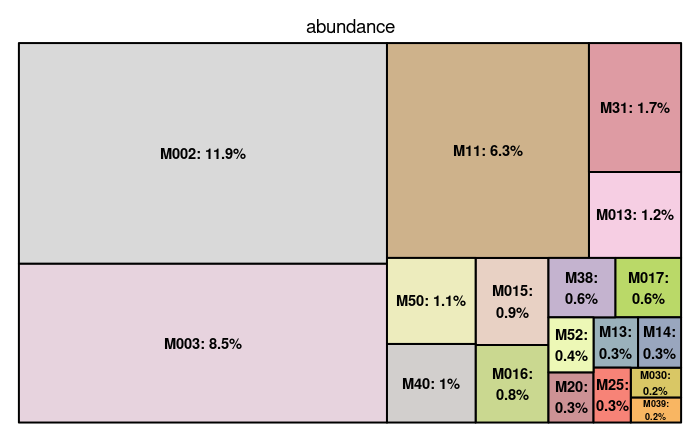


**Supplementary Figure 5.** Coverage of each MAG calculated by read mapping.


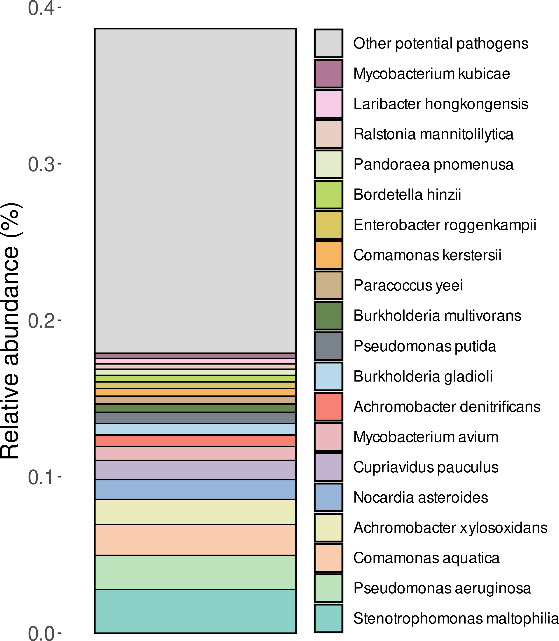


**Supplementary Figure 6.** The presence of possible pathogenic organisms, classified within risk group 2 or higher in the Leibniz Institute DSMZ, in the biofilm. The potential pathogens constituted less than 0.4% of the microbial community.

**References:**

1. Seo, L., Park, S. & Lee, G.-H. Microbiological water quality of water purifiers at elementary schools in Gunsan Area. *The Korean Journal of Microbiology* **45**, 74–81 (2009).

2. Benardini, J. *et al.* International Space Station Internal active thermal control system: an initial assessment of the microbial communities within fluid from ground support and flight hardware. *SAE transactions* 643–650 (2005).

3. Kong, H. H. *et al.* Temporal shifts in the skin microbiome associated with disease flares and treatment in children with atopic dermatitis. *Genome research* **22**, 850–859 (2012).

4. US Food and Drug Administration. FDA database for regulatory grade microbial sequences (FDA-ARGOS): supporting development and validation of infectious disease Dx tests. (2013).

5. Ponpandian, L. N. *et al.* Phylogenetic characterization of bacterial endophytes from four Pinus species and their nematicidal activity against the pine wood nematode. *Scientific Reports* **9**, 12457 (2019).

6. Gu, S. *et al.* Competition for iron drives phytopathogen control by natural rhizosphere microbiomes. *Nature Microbiology* **5**, 1002–1010 (2020).

7. Dutta, D. & Ghosh, K. Screening of extracellular enzyme-producing and pathogen inhibitory gut bacteria as putative probiotics in mrigal, *Cirrhinus mrigala* (Hamilton, 1822). *International Journal of Fisheries and Aquatic Studies* **2**, 310–318 (2015).

8. Füstös, Z., Belák, Á. & Maráz, A. Colonization ability of *Escherichia coli* and *Listeria monocytogenes* in the endosphere of sweet pepper (*Capsicum annuum var. grossum*). *Acta Alimentaria* **46**, 481–491 (2017).

9. Ivanov, V., Stabnikov, V. & Tay, J. H. Removal of the recalcitrant artificial sweetener sucralose and its by-products from industrial wastewater using microbial reduction/oxidation of iron. *ChemEngineering* **2**, 37 (2018).

10. Grice, E. A. *et al.* Topographical and temporal diversity of the human skin microbiome. *science* **324**, 1190–1192 (2009).

11. Zhang, L., Gao, G., Tang, X. & Shao, K. Impacts of different salinities on bacterial biofilm communities in fresh water. *Can. J. Microbiol.* **60**, 319–326 (2014).

12. Im, W. & Lee, S. Comparative analysis of bacterial diversity in the soil of the ginseng field by molecular and cultivation-based technique. *Submission NCBI, accession: AB245383* (2005).

13. Ulrich, K., Becker, R., Behrendt, U., Kube, M. & Ulrich, A. A comparative analysis of ash leaf-colonizing bacterial communities identifies putative antagonists of Hymenoscyphus fraxineus. *Frontiers in Microbiology* **11**, 966 (2020).

14. Karlov, D. S. *et al.* Microbial communities within the water column of freshwater Lake Radok, East Antarctica: predominant 16S rDNA phylotypes and bacterial cultures. *Polar Biology* **40**, 823–836 (2017).

15. Tsiamis, G. *et al.* Olive-mill wastewater bacterial communities display a cultivar specific profile. *Current Microbiology* **64**, 197–203 (2012).

16. Conville, P. S., Murray, P. R. & Zelazny, A. M. Evaluation of the integrated database network system (IDNS) SmartGene software for analysis of 16S rRNA gene sequences for identification of Nocardia species. *Journal of clinical microbiology* **48**, 2995–2998 (2010).

17. Chumpitaz-Segovia, C., Alvarado, D., Ogata-Gutiérrez, K. & Zúñiga-Dávila, D. Bioprospection of native psychrotolerant plant-growth-promoting rhizobacteria from Peruvian Andean Plateau soils associated with *Chenopodium quinoa*. *Canadian Journal of Microbiology* **66**, 641–652 (2020).

18. Sproer, C. *et al.* FDA database for regulatory grade microbial sequences (FDA-ARGOS): Supporting development and validation of Infectious Disease tests. (2021).

19. Vedler, E. *et al.* Limnobacter spp. as newly detected phenol-degraders among Baltic Sea surface water bacteria characterised by comparative analysis of catabolic genes. *Systematic and Applied Microbiology* **36**, 525–532 (2013).

20. Vaïtilingom, M. *et al.* Long-term features of cloud microbiology at the puy de Dôme (France). *Atmospheric environment* **56**, 88–100 (2012).

21. Dhaouadi, S. *et al.* Genome sequences of plant-associated *rhodococcus sp.* isolates from Tunisia. *Microbiology Resource Announcements* **9**, 10–1128 (2020).

22. Zhu, Z. *et al.* Effects of interspecific interactions on biofilm formation potential and chlorine resistance: Evaluation of dual-species biofilm observed in drinking water distribution systems. *Journal of Water Process Engineering* **38**, 101564 (2020).

23. Gray, C. J. & Engel, A. S. Microbial diversity and impact on carbonate geochemistry across a changing geochemical gradient in a karst aquifer. *The ISME Journal* **7**, 325–337 (2013).

24. Delmotte, N. *et al.* Community proteogenomics reveals insights into the physiology of phyllosphere bacteria. *Proceedings of the National Academy of Sciences* **106**, 16428–16433 (2009).

25. Tsuji, M., Tanabe, Y., Vincent, W. F. & Uchida, M. *Vishniacozyma ellesmerensis* sp. nov., a psychrophilic yeast isolated from a retreating glacier in the Canadian High Arctic. *International journal of systematic and evolutionary microbiology* **69**, 696–700 (2019).

26. Nelson, J. & Shaw, A. J. Exploring the natural microbiome of the model liverwort: fungal endophyte diversity in Marchantia polymorpha L. *Symbiosis* **78**, 45–59 (2019).

27. Hirose, D. *et al.* Abundance, richness, and succession of microfungi in relation to chemical changes in Antarctic moss profiles. *Polar Biology* **40**, 2457–2468 (2017).

28. Iliushin, V. A., Kirtsideli, I. Y. & Vlasov, D. Y. Diversity of culturable microfungi of coal mine spoil tips in Svalbard. *Polar Science* **32**, 100793 (2022).

29. Zhang, Z. *et al.* Reconstruction of the paleotemperature in the southern margin of the Taklimakan Desert based on carbon isotope discrimination of Tamarix leaves. *Applied Ecology & Environmental Research* **15**, (2017).

30. Adam, C. & Magdalena, Ś. Species diversity of mycoplankton on the background of selected indicators of water quality in stratified mesotrophic lakes. *International Journal of Environmental Research and Public Health* **19**, 13298 (2022).

31. Solanki, M. K. *et al.* Shifts in the composition of the microbiota of stored wheat grains in response to fumigation. *Frontiers in microbiology* **10**, 1098 (2019).

32. Malli Mohan, G. B. *et al.* Microbiome and metagenome analyses of a closed habitat during human occupation. *MSystems* **5**, e00367-20 (2020).

33. Alfonzo, A., Sicard, D., Di Miceli, G., Guezenec, S. & Settanni, L. Ecology of yeasts associated with kernels of several durum wheat genotypes and their role in co-culture with *Saccharomyces cerevisiae* during dough leavening. *Food Microbiology* **94**, 103666 (2021).

34. Falbesoner, J. Seasonal dynamics of earliest successional fungi in the glacier forefield: isolation and identification from in-growth mesh bags. (2018).

35. Kleinteich, J. *et al.* Pole-to-pole connections: similarities between Arctic and Antarctic microbiomes and their vulnerability to environmental change. *Frontiers in Ecology and Evolution* **5**, 137 (2017).

36. Keshri, J., Pradeep Ram, A. S. & Sime-Ngando, T. Distinctive patterns in the taxonomical resolution of bacterioplankton in the sediment and pore waters of contrasted freshwater lakes. *Microbial Ecology* **75**, 662–673 (2018).

37. von Hoyningen-Huene, A. J. E. *et al.* Bacterial succession along a sediment porewater gradient at Lake Neusiedl in Austria. *Scientific Data* **6**, 163 (2019).

38. Perrin, Y., Bouchon, D., Delafont, V., Moulin, L. & Héchard, Y. Microbiome of drinking water: A full-scale spatio-temporal study to monitor water quality in the Paris distribution system. *Water Research* **149**, 375–385 (2019).

39. Raghupathi *et al.* Microbial diversity and putative opportunistic pathogens in dishwasher biofilm communities. *Applied and Environmental Microbiology* **84**, e02755-17 (2018).

40. Van der Heyden, C. *et al.* Long‐term microbial community dynamics at two full‐scale biotrickling filters treating pig house exhaust air. *Microbial biotechnology* **12**, 775–786 (2019).

41. Bowers, R. M. *et al.* Minimum information about a single amplified genome (MISAG) and a metagenome-assembled genome (MIMAG) of bacteria and archaea. *Nature biotechnology* **35**, 725–731 (2017).

42. Pearson, W. R. An introduction to sequence similarity (“homology”) searching. *Current protocols in bioinformatics* **42**, 3.1. 1-3.1. 8 (2013).

43. Ciric, S., Knezevic, P. & Petrovic, O. Low nutrient R2A medium in the assessment of bacteriological status of water and other environments. *Environment* **1**, 2 (2022).

44. Sagen, A. Lysogeny Broth (LB) medium. (2023).

45. Satari, L., Guillén, A., Vidal-Verdú, À. & Porcar, M. The wasted chewing gum bacteriome. *Scientific Reports* **10**, 16846 (2020).

46. Satari, L. *et al.* *Sagittula salina* sp. nov., isolated from marine waste. *International Journal of Systematic and Evolutionary Microbiology* **72**, (2022).

47. Sohlberg, E., Sarlin, T. & Juvonen, R. Fungal diversity on brewery filling hall surfaces and quality control samples. *Yeast* **39**, 141–155 (2022).

48. Feng, L., Gu, J., Guo, L., Mu, G. & Tuo, Y. Safety evaluation and application of lactic acid bacteria and yeast strains isolated from Sichuan broad bean paste. *Food Science & Nutrition* **11**, 940–952 (2023).

49. Sheet, P. D. Tmp 001–brain heart infusion agar plate.

50. Vasmara, C., Marchetti, R. & Carminati, D. Wastewater from the production of lactic acid bacteria as feedstock in anaerobic digestion. *Energy* **229**, 120740 (2021).
